# 7-Methoxyisoflavone ameliorates atopic dermatitis symptoms by regulating multiple signaling pathways and reducing chemokine production

**DOI:** 10.1038/s41598-022-12695-3

**Published:** 2022-05-24

**Authors:** Hao Dong, Chenjie Feng, Xiyunyi Cai, Yuanyuan Hao, Xinyue Gu, Lei Cai, Shuting Wu, Jiamin Chen, Zhou Liu, Wen Xie, Xuanren Lu, Hongfa Qian, Yulin Liu, Yiming Cao, Junlin Zhu, Jiayi Xu, Yanjie Zhou, Shuangyu Ma, Sha Yang, Yufeng Shi, Haojiang Yu, Minjie Shi, Yurong Wang, Harvest F. Gu, Lei Fan, Liang Wu

**Affiliations:** 1 Jiangsu Key Laboratory of Drug Screening, Institute of Pharmaceutical Sciences, China Pharmaceutical University, Nanjing, 210009 People’s Republic of China; 2 School of Life Science and Technology, China Pharmaceutical University, Nanjing, 210009 People’s Republic of China; 3 Department of Applied Mathematics and Statistics, The Johns Hopkins University, Baltimore, MD 21218 USA; 4 School of Pharmacy, China Pharmaceutical University, Nanjing, 210009 People’s Republic of China; 5 School of Traditional Chinese Pharmacy, China Pharmaceutical University, Nanjing, 210009 People’s Republic of China; 6 School of Basic Medicine and Clinical Pharmacy, Center for Pathophysiology, China Pharmaceutical University, Nanjing, 210009 People’s Republic of China; 7 Department of Hematology, Jiangsu Province Hospital, The First Affiliated Hospital of Nanjing Medical University, Collaborative Innovation Center for Cancer Personalized Medicine, Nanjing, 210009 People’s Republic of China

**Keywords:** Drug discovery, Immunology

## Abstract

7-Met, a derivative of soybean isoflavone, is a natural flavonoid compound that has been reported to have multiple signaling pathways regulation effects. This study investigated the therapeutic effects of 7-Met on mice with atopic dermatitis induced by fluorescein isothiocyanate (FITC), or oxazolone (OXZ). 7-Met ameliorated FITC or OXZ-induced atopic dermatitis symptoms by decreasing ear thickness, spleen index, mast cell activation, neutrophil infiltration and serum IgE levels in female BALB/c mice. In FITC-induced atopic dermatitis mice, 7-Met reduced Th1 cytokines production and regulated Th1/Th2 balance by downregulating the secretion of thymic stromal lymphopoietin (TSLP) via inactivation of the NF-κB pathway. In OXZ-induced atopic dermatitis, 7-Met functioned through the reduction of Th17 cytokine production. Our study showed that 7-Methoxyisoflavone alleviated atopic dermatitis by regulating multiple signaling pathways and downregulating chemokine production.

## Introduction

Atopic dermatitis (AD) is a commonly encountered allergic inflammatory skin disease influenced by multiple environmental factors (e.g., mite, dust, smoking, pollen, exposure to allergens, etc.)^1^. It affects 15–20% of children and 1–3% of adults worldwide^2–4^. Clinical hallmarks of AD include skin redness, itching, peeling and skin hypersensitivity^5^. Histological examination reveals inflammatory infiltrates consisting of T cells, monocyte, neutrophil and mast cell. As a vital player in AD, Mast cell regulate eosinophil activation and recruitment^6^, and produce histamine and other inflammatory mediators contributing to itching and inflammation^7^.

The development and pathophysiology of AD are multifactorial. From an immune balance perspective, AD results from an imbalance of T cells, particularly T helper cell types 1 (Th1 cells) and 2 (Th2 cells)^8^. Th1 cells are defined by the expression of interferon (IFN) γ and the signature transcription factor T-bet participates in type 1 immune responses^9^. Th2 cells, induced by environmental factors, or allergens, produce Th2 cytokines such as interleukin (IL)-4^10^, IL-5^8^ and IL-13^11^, which are regarded to be the main player of AD. Current studies found that T helper 17 cells (Th17 cell) and their cytokines in peripheral blood from AD patients were highly correlated with the severity of AD^12^. Upon activation of STAT3, Th17 cells activate and recruit neutrophils which produce chemokines in a p38 MAPK-dependent manner^13–15^.

Atopic dermatitis is regulated primarily by T cells within the adaptive immune system, as well as by natural killer and innate lymphoid cells within the innate immune system. The chemokine receptor system, consisting of chemokine peptides and chemokine G protein–coupled receptors^16^, is a critical regulator of inflammatory processes in AD^17^. Further studies have found that the differences in the chemotactic response could modify the T migratory response of the different T-cell populations. IFN-γ, the classic Th1-polarizing cytokine, increases the expression of Cxcr3 and its ligands C–X–C motif chemokine ligand (Cxcl)9, Cxcl10 and Cxcl11^18–20^. C–C motif chemokine ligand (Ccl)22 is known as a macrophage-derived chemokine and is a Th2 response–associated chemokine. Ccl17 is known as a thymus activation-regulated chemokine. Similar to Ccl22, the expression of Ccl17 is regulated by Th2 cells cytokines, such as IL-4^20,21^. IL-17 is produced by Th17 cells and induces the production of chemokines such as Cxcl1 and Cxcl2^22^. Modulation of chemokines provides an attractive therapeutic target for AD.

Fluorescein isothiocyanate (FITC) and oxazolone (OXZ) are two haptens frequently used independently to establish AD models^23,24^. Initial topical sensitization of mice to FITC resulted in increased IgE levels, as well as the development of FITC-specific Th1 cells^25^. Thymic stromal lymphopoietin (TSLP) was originally identified in a murine thymic stromal cell line as a lymphoid growth factor^26^. TSLP-activated DCs up-regulate surface OX40-ligand expression and down-regulate IL-12 production, preferentially promoting naive CD4^+^ T cells’ differentiation into the Th2 phenotype^27^. Larson et al.^28^ found that following FITC sensitization and challenging, TSLP receptor-deficient mice exhibited a dramatically reduced allergic response, confirming that TSLP is required for the development of Th2 dominated response induced by FITC in combination with dibutyl phthalate as a sensitizing agent^25^. OXZ has been thought to induce a Th1 dominated response^29^. However, studies have shown that skin inflammation was changed from a typical Th1 dominated delayed-type hypersensitivity response to a chronic Th2 dominated inflammatory response when hairless mice were multiply challenged with OXZ^30^.

Current treatment for AD includes oral antihistamines, steroids and calcineurin inhibitors^31,32^. However, antihistamines can lead to considerable side effects including sedation and psychomotor retardation. Long-term usage of inhaled steroids is often accompanied by undesirable adverse effects such as acne and skin atrophy. Thus, it is necessary to find effective novel therapeutic agents with fewer side effects. Studies have shown that isoflavones^33^ can reduce the activation of MAPK induced by IL-22, IL-17A, and TNF-α in normal human epidermal keratinocytes. Shin et al.^34^ showed that 7-Methoxyisoflavone (7-Met), as a type of isoflavones derivatives, has a higher NF-κB-inhibition activity than most of the normal flavones in HCT116 cell. Hence, we speculate that 7-Met might have a better pharmacological activity than flavones in AD treatment. In the current study, we investigated the effect of 7-Met in FITC or OXZ-induced AD models and explored the mechanisms.

## Results

### 7-Methoxyisoflavone alleviated skin inflammation in FITC/OXZ-induced AD models

**Figure 1:**
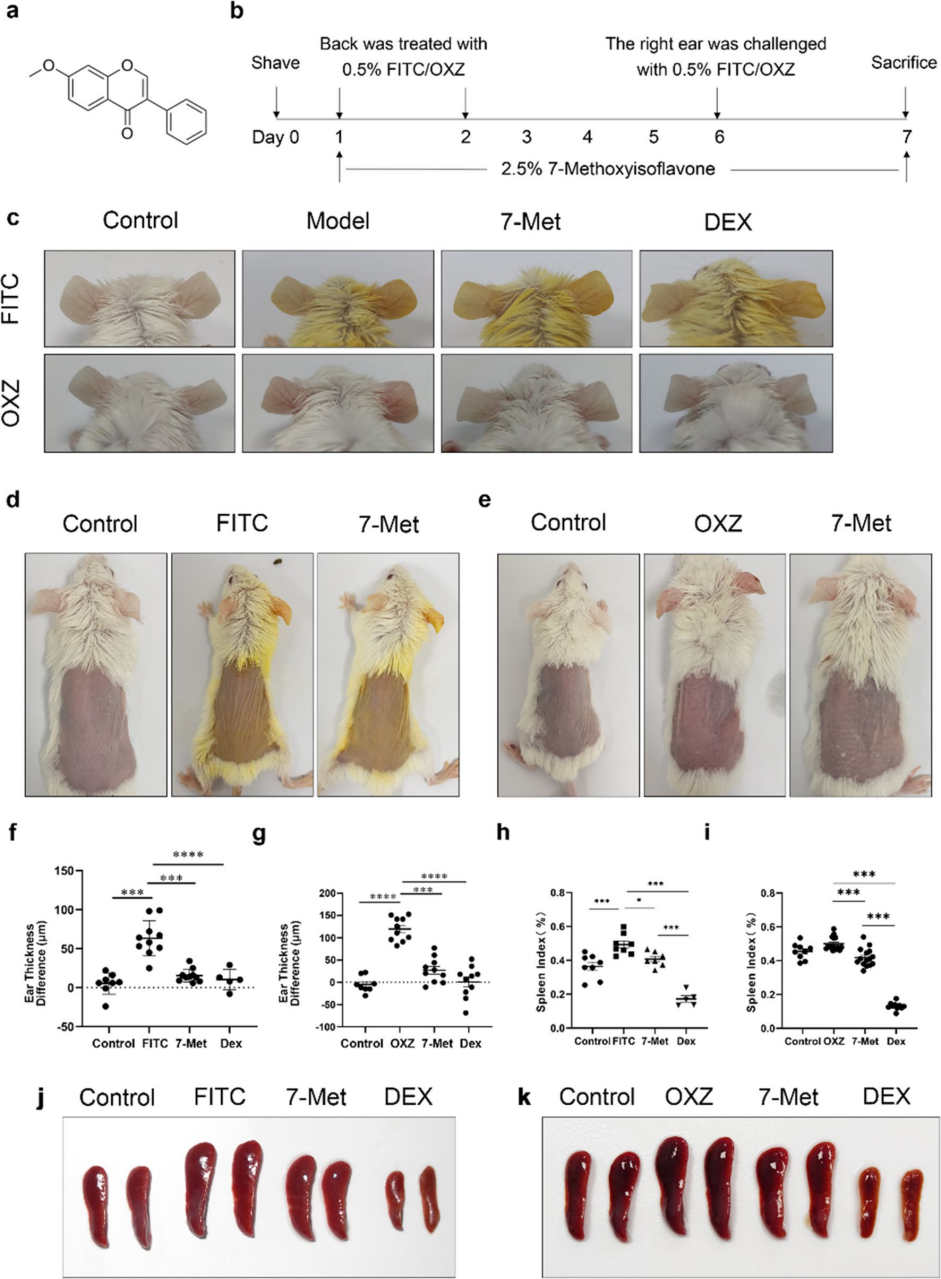
Experimental design and therapeutic effect of 7-Methoxyisoflavone in AD mice. (**a**) Structure of 7-Methoxyisoflavone (7-Met). (**b**) Experimental design for the induction of Atopic dermatitis (AD). (**c**) Clinical features of the ears appearance are shown. (**d**) Whole-body photos of atopic dermatitis mice induced by FITC. (These photos were taken by Yuanyuan Hao and Hongfa Qian). (**e**) Whole-body photos of atopic dermatitis mice induced by OXZ (These photos were taken by Yuanyuan Hao and Hongfa Qian). (**f**) Effect of the 7-Met on ear thickness induced by FITC. (p_FITC_ < 0.0001, p_7-Met_ < 0.0001, p_DEX_ < 0.0001, one-way ANOVA). (**g**) Effect of the 7-Met on ear thickness induced by OXZ. (p_OXZ_ < 0.0001, p_7-Met_ < 0.0001, p_DEX_ < 0.0001, one-way ANOVA). (**h**) Effect of the 7-Met on spleen index induced by FITC. The spleen index of mice treated with 7-Met was as significantly reduced (p = 0.0083, two-tailed). (**i**) Effect of the 7-Met on spleen index induced by OXZ. The spleen index of mice treated with 7-Met was as significantly reduced (p < 0.0001, one-way ANOVA). (**j**) Clinical features of spleens induced by FITC. (**k**) Clinical features of spleens induced by OXZ. Values are expressed as the means ± S.E.M. (n = 5–10). *p < 0.05, **p < 0.01, ***p < 0.001 and ****p < 0.0001. *FITC* fluorescein isothiocyanate, *OXZ* oxazolone, *7-Met* 7-Methoxyisoflavone, *DEX* dexamethasone.

To investigate the effect of 7-Met on AD-like skin inflammation, we induced AD mice model with 0.5% FITC or 0.5% OXZ independently. These mice were treated with or without 2.5% 7-Met (Fig. 1a) for 1 week (Fig. 1b). As indicated in Fig. 1c, FITC or OXZ induced remarkable AD lesions such as erythema, ear thickening, hemorrhage, edema, excoriation and scaling, all of which were diminished by treatment of 7-Met or the positive control, dexamethasone (DEX). Notably, although the ear thickness of mice treated with 7-Met was as significantly reduced as those treated with DEX (Fig. 1d,e), no significant decrease of the spleen index (Fig. 1f–i) was observed in the 7-Met treated groups compared to the DEX treated group, which showed severe immunosuppression as indicated by a remarkable reduction of the spleen index (Fig. 1j,k).

### 7-Methoxyisoflavone suppressed epidermis thickening and mast cell infiltration

**Figure 2:**
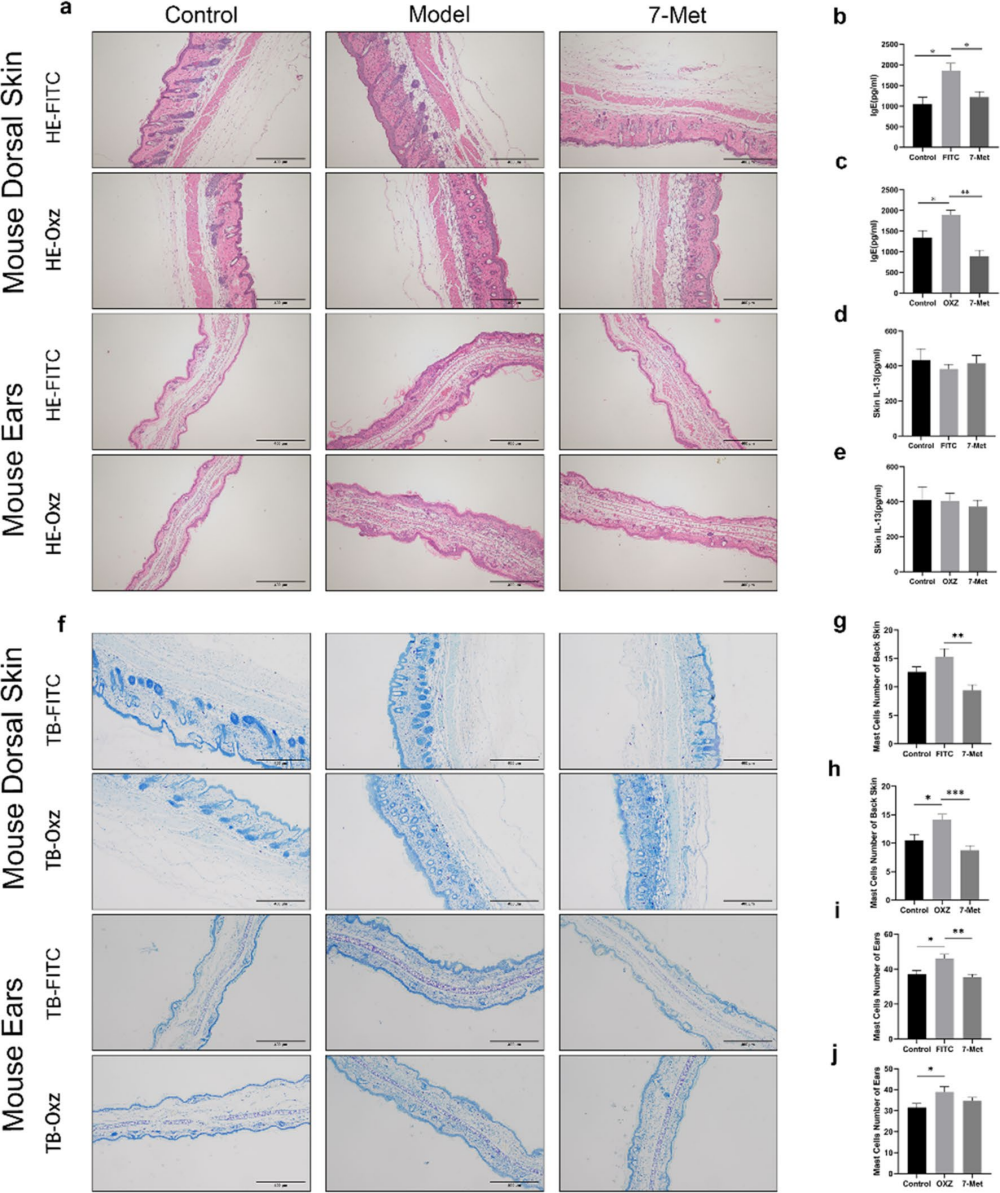
Histological analysis of AD mice. (**a**) Representative H&E-stained (scale bar = 400 μm) cross sections of ears and dorsal skin. (**b**) Total serum IgE level of FITC-model was measured by enzyme-linked immunosorbent assay (ELISA) (p_Model_ = 0.0127, p_7-Met_ = 0.0159). Each bar represents the mean ± S.E.M. of ten independent experiments. (**c**) Total serum IgE level of OXZ-model was measured by enzyme-linked immunosorbent assay (ELISA) (p_Model_ = 0.0367, p_7-Met_ = 0.0061). (**d**) Skin IL-13 level of FITC-model was measured by enzyme-linked immunosorbent assay (ELISA). (**e**) Skin IL-13 level of OXZ-model was measured by enzyme-linked immunosorbent assay (ELISA). (**f**) Representative TB-stained (scale bar = 200 μm) cross sections of ears and dorsal skin. (**g**) The number of mast cells in mouse dorsal skin treated by FITC or 7-Met was measured using ImageJ software. Values are expressed as the means ± S.E.M. (n = 3). (p_7-Met_ = 0.0011). (**h**) The number of mast cells in mouse dorsal skin treated by OXZ or 7-Met was measured using ImageJ software. Values are expressed as the means ± S.E.M. (n = 3). (p_Model_ = 0.0201, p_7-Met_ = 0.0003). (**i**) The number of mast cells in mouse ears treated by FITC or 7-Met was measured using ImageJ software. Values are expressed as the means ± S.E.M. (n = 3). (p_Model_ = 0.0108, p_7-Met_ = 0.0019). (**j**) The number of mast cells in mouse ears treated by OXZ or 7-Met was measured using ImageJ software. Values are expressed as the means ± S.E.M. (n = 3). (p_Model_ = 0.0362) Each bar represents the mean ± S.E.M. of ten independent experiments. ^#^p < 0.05, ^##^p < 0.01 VS FITC. *p < 0.05, **p < 0.01 VS OXZ. *FITC* fluorescein isothiocyanate, *OXZ* oxazolone, *7-Met* 7-Methoxyisoflavone, *DEX* dexamethasone, *H&E* hematoxylin and eosin, *TB* toluidine blue.

Mast cells are the dominant effector cells involved in the atopic dermatitis and they secrete a variety of bioactive substances such as histamine and pro-inflammatory cytokines to lead to epidermal thickening. To further determine the effect of 7-Met on skin epidermal hyperplasia and mast cell infiltration, the ear sections and dorsal skin sections were stained by hematoxylin and eosin (H&E)/Toluidine Blue (TB) and examined under an optical microscope. In agreement with the phenotypic observation, repeated FITC/OXZ exposure caused potent inflammatory changes, such as prominently epidermal hyperplasia, inflammation of the dermis with reference to the control group (Fig. 2a). Elevated IgE level, the typical symptom of AD, was observed in the model group but decreased in the 7-Met group (Fig. 2b,c). However, IL-13 levels in the mouse skin did not change significantly. (Fig. 2d,e).

The application of 7-Met attenuated FITC/OXZ-induced AD-like histopathological signs on the ear significantly. We next examined the numbers and distribution of mast cells through Toluidine Blue staining in AD-like mice (Fig. 2f) and the effects of 7-Met on the infiltration of mast cells were explored. As shown in Fig. 2g–j, the mast cells in ears in the model group increased compared to those in the control group, whereas the administration of 7-Met decreased mast cell infiltration.

**Figure 3:**
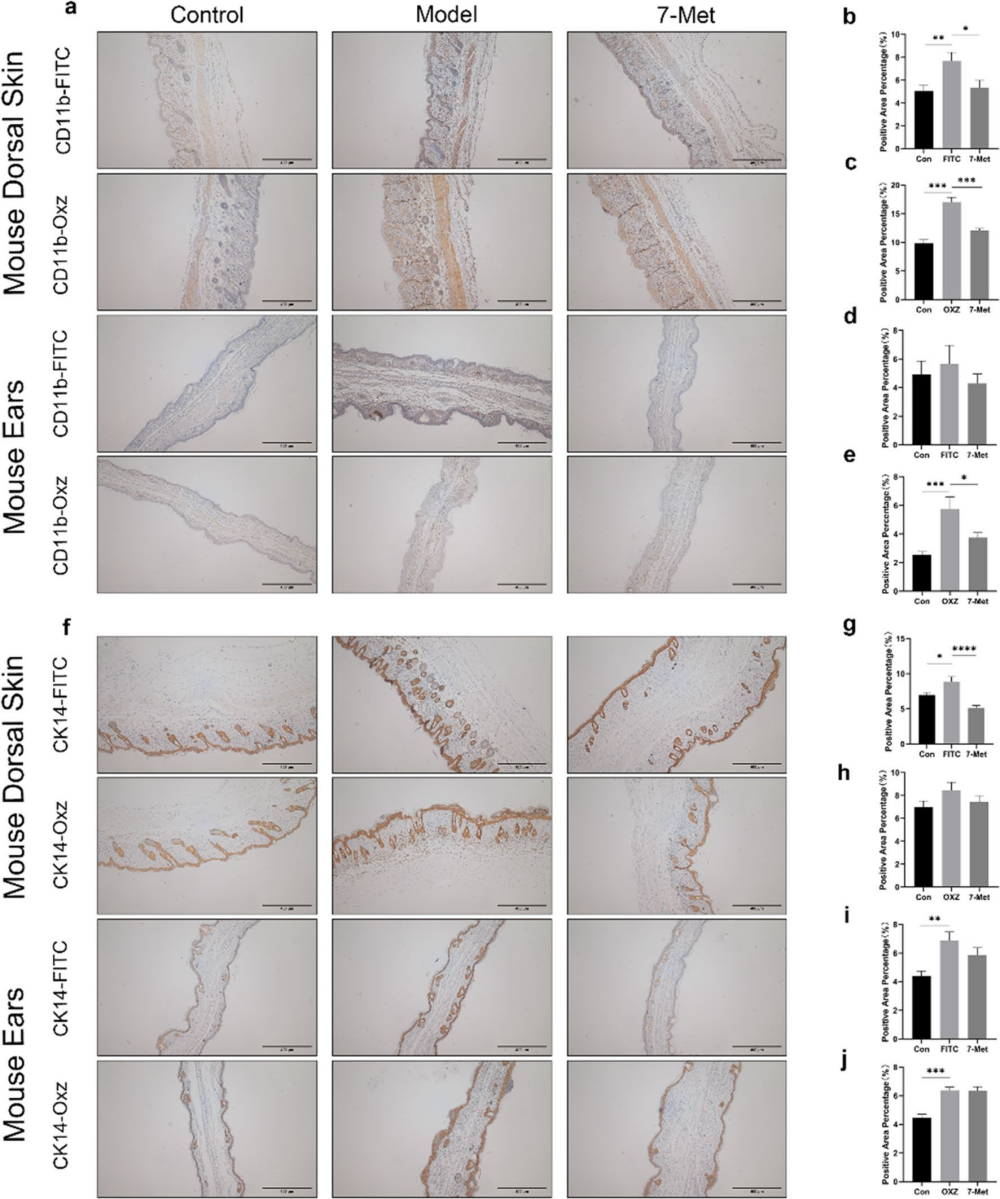
Regulatory effects of drugs on neutrophils, macrophages and keratinocytes in AD mice. (**a)** Representative CD11b immunohistochemistry-stained (scale bar = 400 μm) cross sections of ears and dorsal skin. (**b**) The quantitative analysis of positive area in mouse dorsal skin treated by FITC or 7-Met was carried out using ImageJ software. Values are expressed as the means ± S.E.M. (n = 3). (p_Model_ = 0.0072, p_7-Met_ = 0.0126). (**c**) The quantitative analysis of positive area in mouse dorsal skin treated by OXZ or 7-Met was carried out using ImageJ software. Values are expressed as the means ± S.E.M. (n = 3). (p_Model_ < 0.0001, p_7-Met_ < 0.0001). (**d**) The quantitative analysis of positive area in mouse ears treated by FITC or 7-Met was carried out using ImageJ software. Values are expressed as the means ± S.E.M. (n = 3). (**e**) The quantitative analysis of positive area in mouse ears treated by OXZ or 7-Met was carried out using ImageJ software. Values are expressed as the means ± S.E.M. (n = 3). (p_Model_ = 0.0003, p_7-Met_ = 0.0352). (**f**) Representative CK14 immunohistochemistry-stained (scale bar = 200 μm) cross sections of ears and dorsal skin. (**g**) The quantitative analysis of positive area in mouse dorsal skin treated by FITC or 7-Met was carried out using ImageJ software. Values are expressed as the means ± S.E.M. (n = 3). (p_Model_ = 0.0183, p_7-Met_ < 0.0001). (**h**) The quantitative analysis of positive area in mouse dorsal skin treated by FITC or 7-Met was carried out using ImageJ software. Values are expressed as the means ± S.E.M. (n = 3). (**i**) The quantitative analysis of positive area in mouse ears treated by FITC or 7-Met was carried out using ImageJ software. Values are expressed as the means ± S.E.M. (n = 3). (p_Model_ = 0.0024). (**j**) The quantitative analysis of positive area in mouse ears treated by FITC or 7-Met was carried out using ImageJ software. Values are expressed as the means ± S.E.M. (n = 3). (p_Model_ < 0.0001). Values are expressed as the means ± S.E.M. (n = 3). (p_Model_ = 0.0362) Each bar represents the mean ± S.E.M. of ten independent experiments. ^#^p < 0.05, ^##^p < 0.01 VS FITC. *p < 0.05, **p < 0.01 VS OXZ. *FITC* fluorescein isothiocyanate, *OXZ* oxazolone, *7-Met* 7-Methoxyisoflavone, *DEX* dexamethasone, *H&E* hematoxylin and eosin, *TB* toluidine blue.

Neutrophils and keratinocytes play an irreplaceable role in the pathogenesis of common chronic inflammatory skin diseases such as psoriasis and atopic dermatitis, characterized by infiltration of neutrophil and hyperproliferation of keratinocyte. To determine the effect of 7-Met on neutrophil infiltration, the ear sections and dorsal skin sections were stained by CD11b immunohistochemistry and examined under an optical microscope. In agreement with the phenotypic observation, repeated FITC/OXZ exposure resulted in prominent neutrophil infiltration compared to the control group (Fig. 3a). As illustrated in Fig. 3b–e, the neutrophil infiltration in the model group increased compared to the control group, while the administration of 7-Met decreased neutrophil infiltration.

Keratinocytes are the major cell population of the epidermis and can be activated to produce various chemokines. Next, the keratinocyte proliferation of AD mice was examined through CK14 staining in AD-like mice (Fig. 3f) and the effects of 7-Met on the proliferation of keratinocytes were explored. Figure 3g–j implied that the keratinocytes in ears in the model group increased compared to the control group, while the administration of 7-Met decreased epidermal thickness.

To sum up, 7-Met administration attenuated the inflammation of the epidermal, infiltration of mast cells and epidermis thickness.

### Different expression profile of 7-Methoxyisoflavone in FITC/OXZ-induced AD models

Based on our observations, it is clear that mast cells act early in response to cutaneous allergen exposure. To identify these changes caused by mast cells or other inflammatory cells, we used RNA-seq followed by Kyoto Encyclopedia of Genes and Genomes (KEGG)^35^ and Gene Ontology (GO)^36,37^ analysis to study global changes in gene expression in control and AD mice. For both models, we identified genes whose expression was significantly induced or repressed in the haptens-treated group compared to the control group, filtered the lists to those with a twofold or greater change (increase or decrease), and performed KEGG pathway and GO analysis independently for the FITC and OXZ-induced models.

**Figure 4:**
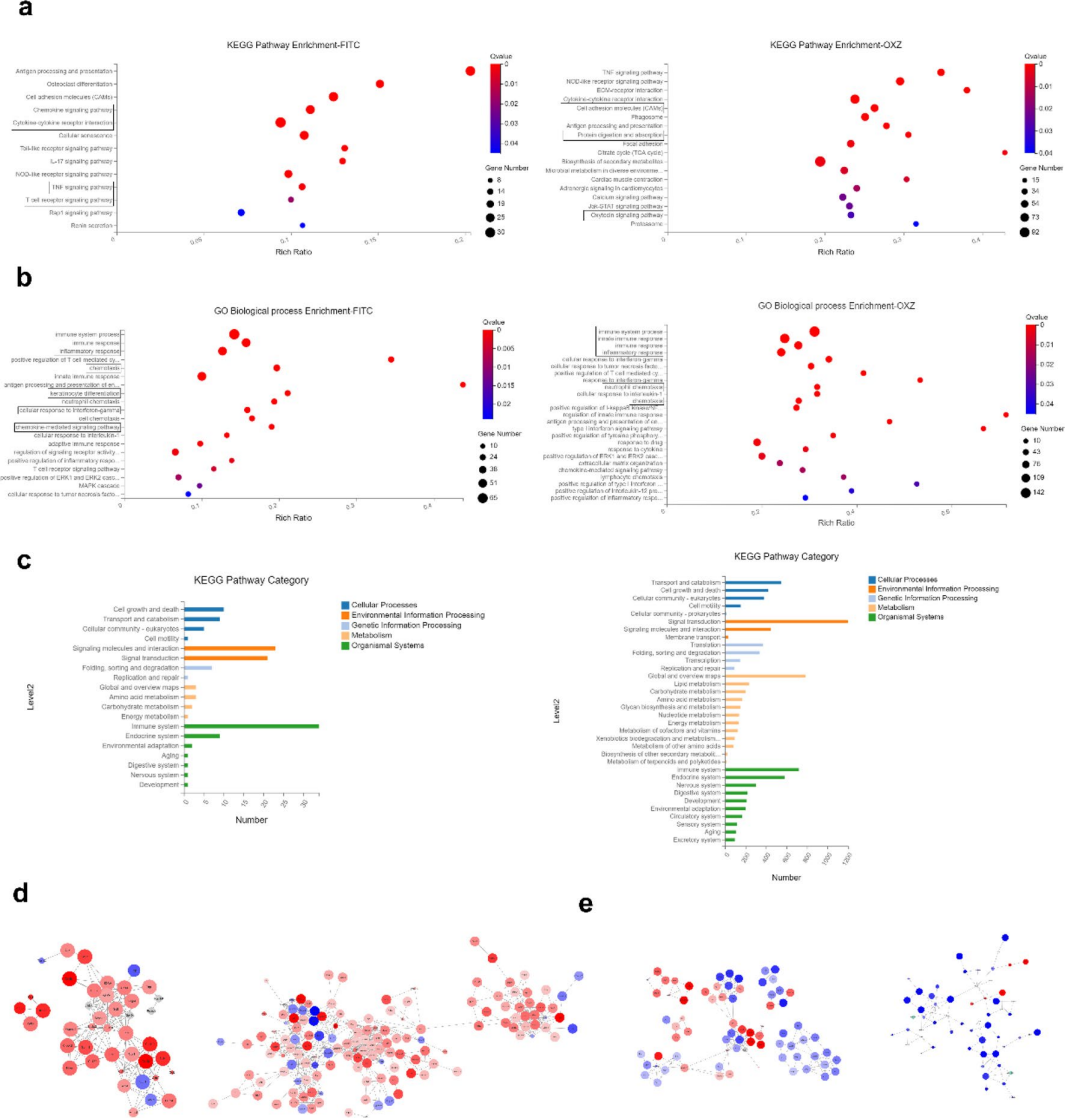
Kyoto Encyclopedia of Genes and Genomes (KEGG) pathway enrichment of different expression genes. (**a**) Kyoto Encyclopedia of Genes and Genomes (KEGG) pathway enrichment of different expression genes. The size of the bubble indicates the number of genes annotated to a particular KEGG Pathway. (**b**) Gene Ontology (GO) Biological Process enrichment of different expression genes. The size of the bubble indicates the number of genes annotated to a particular KEGG Pathway. (**c**) KEGG categories of different expression genes. (**d**) Protein–protein interaction (PPI) networks of predicted DEGs regulated by FITC or OXZ were assembled. Nodes were colored by fold changes between haptens-treated and control groups. (**e**) PPI networks of predicted DEGs in different models regulated by 7-Met were assembled. Nodes were colored by fold changes between 7-Met and haptens-treated groups. All PPI networks were assembled according to STRING database using Cystoscope 3.8.2 and the node size represents the enriched q-value value, the larger the node, the smaller the q-value (Supplementary Table 2).

In FITC-induced model, the primary pathway of genes regulated by 7-Met identified by KEGG and GO enrichment^38^ were related to antigen processing and presentation, responses to IFN-γ, chemokine signaling pathway, cytokine-cytokine receptor interaction and the chemotaxis of multiple categories of cells (Fig. 4a–c). This included upregulation of the pro-inflammatory cytokines (IL-4) and chemokines (Ccl17, Ccl22, Cxcl2) (Fig. 4d,e), suggesting that Th1/Th2 balance may play an important role in FITC-induced AD model. KEGG and GO analysis also identified TNF signaling pathway, immune responses and ERK-MAPK signaling pathway as the key processes affected in the mice following FITC-administration.

The primary network identified by KEGG and GO analysis in OXZ-induced model included genes regulating MAPK, JAK-STAT and Ras signaling pathways. They contained genes that regulate or are regulated by MAPK, NF-κB, or Ras signaling pathways that have all been implicated in mediating atopic dermatitis responses (Fig. 4a–c).

Our analysis identified ERK1/2 signaling pathway and neutrophils chemotaxis as the key mechanism in OXZ-induced AD model. We also observed remarkable up-regulation of chemokines (Cxcl2, Cxcl3, Cxcl5) in OXZ-induced model (Fig. 4d,e). It is important to highlight that the expression of most of the pro-inflammatory genes are down-regulated following the treatment of 7-Met.

### 7-Methoxyisoflavone attenuated FITC-induced AD symptoms by regulating Th1/Th2 balance

**Figure 5:**
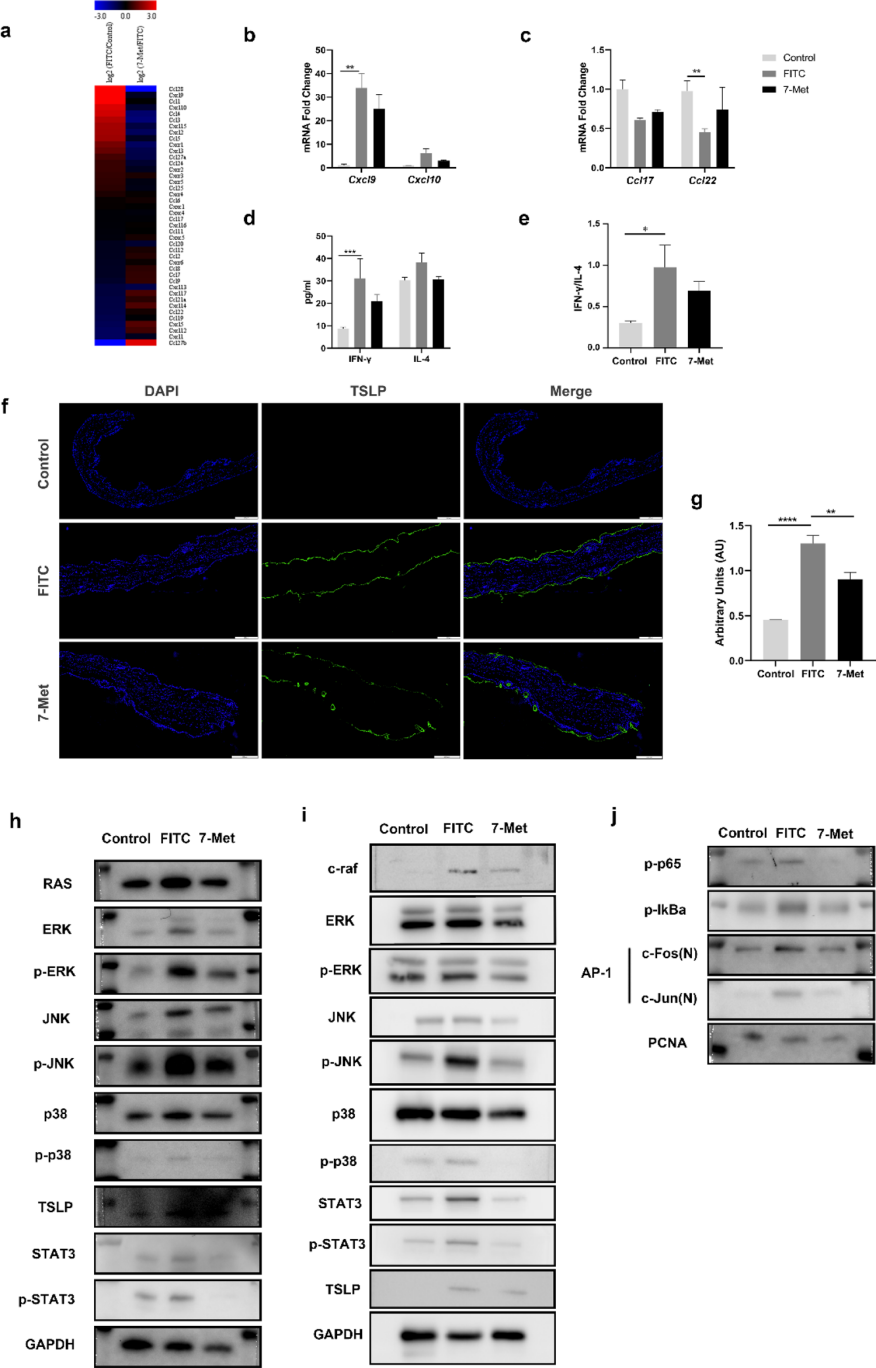
7-Methoxyisoflavone attenuated FITC-induced AD symptoms by regulating Th1/Th2 balance and MAPK-AP1 signaling pathway. (**a**) Heatmap of all significant different chemokines related genes between model (FITC) and drug-treated mice ear tissue. (**b**) Relative Cxcl9 (p = 0.0066), and Cxcl10 mRNA levels were determined by quantitative RT-PCR. (**c**) Relative Ccl17 (p = 0.0096) and Ccl22 (p = 0.0072) mRNA levels were determined by quantitative RT-PCR. (**d**) Total serum IFN-γ and IL-4 levels were measured by enzyme-linked immunosorbent assay (ELISA). (**e**) The ratio of IL-4 to IFN-γ was calculated (p = 0.0318) (n = 10). (**f**) TSLP proteins labelled with FITC fluorescent dye was shown in green. Nuclei labeled with DAPI was shown in blue. (**g**) The average fluorescence intensity was shown as mean ± SEM. (n = 6). (p_model_ < 0.0001, two-tailed, p_7-met_ = 0.0053, two-tailed). (**h**) The phosphorylation of MAPKs and expression levels of TSLP in the ear extracts was analyzed by Western blotting. (**i**) The phosphorylation of MAPKs and STAT3 in the dorsal skin extracts was analyzed by Western blotting. (**j**) The expression levels of p-IκBα, p-p65 (N) and the nuclear translocation of AP-1in the ear extracts were analyzed by Western blotting. The expression levels of Ras, c-Raf, p-ERK, ERK, p-JNK, JNK, p-p38 and p38 in the total protein and the nuclear translocation of c-Jun and c-Fos of the right ears were detected by Western blotting. *FITC* fluorescein isothiocyanate, *7-Met* 7-Methoxyisoflavone, *TSLP* thymic stromal lymphopoietin, *Ccl* C–C motif chemokine ligand, *Cxcl* C-X-C motif chemokine ligand; *p < 0.05, **p < 0.01, ***p < 0.001 and ****p < 0.0001. Interleukin; *IFN* Interferon, *FITC* fluorescein isothiocyanate, *OXZ* oxazolone, *7-Met* 7-Methoxyisoflavone, *p65 (N)* nucleus p65, *p-ERK* phospho-p44/42MAPK(ERK1/2), *ERK* p44/42MAP Kinase, *p-p38* phospho-p38 MAPK(Thr180/Tyr182), *p38* p38 MAPK, *c-Fos (N)* nucleus c-Fos, *c-Jun (N)* nucleus. In order to avoid the interference of non-specific binding of antibodies to chemiluminescence imaging. Under the premise of ensuring credibility, some WB images are cropped strips, all images have been provided in the Supplementary Document.

Based on the KEGG and GO analysis shown in Fig. 5a, chemokine transcriptions were significantly altered in FITC-induced mice model. chemokines were shown to be involved in the development of AD. Cxcl9, Cxcl10 have been identified within a Th1 type of response, whereas Ccl17 and Ccl22 are classically characterized in a Th2 type of response. Therefore, we verified the expression of Th1/Th2 cell-associated chemokines by RT-qPCR.7-Met decreased the transcription of Th1 cell-associated chemokines (Cxcl9 and Cxcl10) and Th2 cell-associated chemokines (Ccl17 and Ccl22) in FITC-induced AD mice model (Fig. 5b,c).

Imbalance in the CD4+ T cell subsets is characteristic of AD as the balance of Th1 and Th2 cells play a vital role in AD by modulating the secretion of Th1-related cytokines (e.g., IFN-γ) and Th2-related cytokines (e.g., IL-4)^9^. We examined serum levels of inflammatory cytokines associated with the Th1/Th2 balance. The IFN-γ and IL-4 levels were elevated in FITC-induced model group while decreased in the 7-Met-treated group (Fig. 5d). Th1/Th2 ratio, determined by serum IFN-γ/IL-4, was slightly decreased by 7-Met (Fig. 5e). These results suggest that 7-Met ameliorated AD symptoms partly through the modulation of Th1/Th2 balance. TSLP is a hematopoietic factor that plays a pivotal role in Th1/Th2 homeostasis^39^. To corroborate the causal link between TSLP and naive CD4^+^ T cells, we examined TSLP expression by Immunofluorescence assay and Western blotting. Our results showed a significant increase in TSLP after treatment with FITC. These increases could be significantly lowered with 7-Met (Fig. 5f–h). NF-κB is critical for inflammation-induced expression of TSLP as an orthologous NF-κB binding site was found 3.7 kb upstream of the promoter of the mouse TSLP gene^40^. To analyze the effect of 7-Met on NF-κB signaling, we examined the phosphorylation of IkBα in FITC-induced model. It turned out that 7-Met significantly inhibited NF-κB signaling in the FITC treated mice as phospho-IkBα and p65 were downregulated (Fig. 5j).

Studies have revealed the critical role of MAPK-AP1 signaling in inflammatory cytokines production^41^. We hypothesized that 7-Met may reduce the production of cytokines involved in AD by targeting the MAPK-AP-1 signaling pathway. Therefore, we used Western blotting to verify the inhibitory effect of 7-Met on MAPK pathway. As expected, 7-Met administration effectively reversed FITC-induced activation of Ras, c-Raf, ERK, JNK and p38 in mice ear tissue and dorsal skin, as indicated by their protein expression and phosphorylation (Fig. 5h,i). Accordingly, 7-Met suppressed AP-1 transcriptional activity in AD mice by decreasing the expression of AP-1 components including c-Fos and c-Jun (Fig. 5j).

### 7-Methoxyisoflavone attenuated OXZ-induced AD symptoms by reducing Th17 cells subset

**Figure 6:**
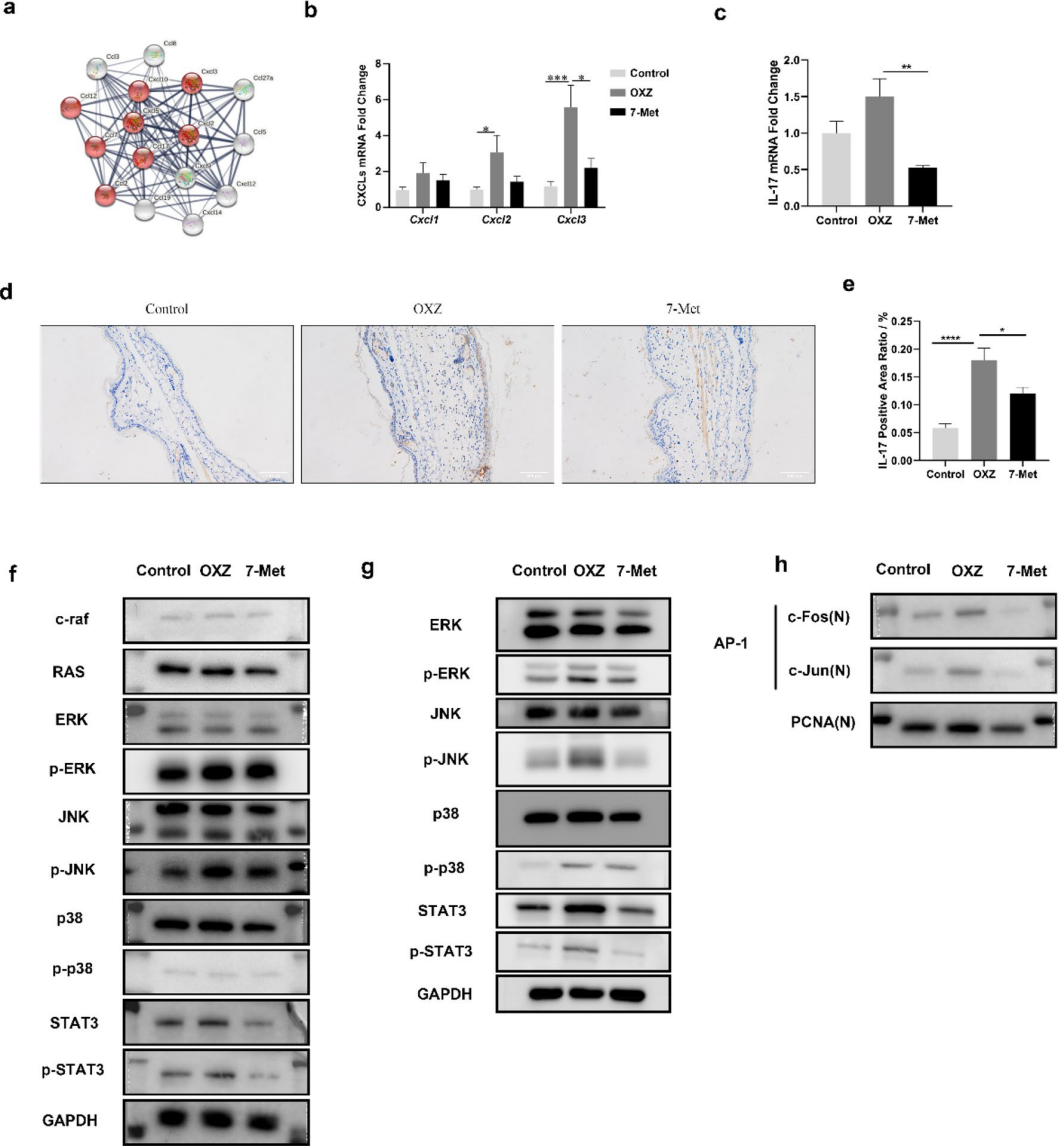
7-Methoxyisoflavone downregulated Th17 cells subset. (**a**) Protein–protein interaction (PPI) network of OXZ-induced differentially expressed (|Foldchange|> 2, q < 0.05) chemokines mRNAs, the nodes in red indicate genes involved in the IL-17 signaling pathway (KEGG: mmu04657, FDR < 0.0001). (**b**) Relative Cxcl1, Cxcl2 and Cxcl3 mRNA levels were determined by quantitative RT-qPCR. (**c**) Relative IL-17A mRNA levels were determined by quantitative RT-PCR (p_7-met_ = 0.0096, two-tailed). (**d**) Immunohistochemistry staining of IL-17A+ Th17 cells. (**e**) IL-17A positive area ratio was calculated by ImageJ software (n = 6). (p_model_ < 0.0001, two-tailed, p_7-met_ = 0.0243). (**f**) The phosphorylation of MAPKs and STAT3 in the ears total protein was analyzed by Western blotting. (**g**) The phosphorylation of MAPKs and STAT3 in the dorsal skin total protein was analyzed by Western blotting. (**h**) The nuclear translocation of AP-1 was analyzed by Western blotting. The expression levels of Ras, c-Raf, p-ERK, ERK, p-JNK, JNK, p-p38 and p38 in the total protein and the nuclear translocation of c-Jun and c-Fos of the right ears were detected by Western blotting. *p < 0.05 and ****p < 0.0001. *p-STAT3* phospho-STAT3(Tyr705); *STAT3*, STAT3(124H6). *7-Met* 7-Methoxyisoflavone, *p65 (N)* nucleus p65, *p-ERK* phospho-p44/42MAPK(ERK1/2), *ERK* p44/42MAP Kinase, *p-p38* phospho-p38 MAPK (Thr180/Tyr182), *p38*, p38 MAPK, *c-Fos (N)* nucleus c-Fos, *c-Jun (N)* nucleus. In order to avoid the interference of non-specific binding of antibodies to chemiluminescence imaging. Under the premise of ensuring credibility, some WB images are cropped strips, all images have been provided in the Supplementary Document.

To explore the underlying therapeutic effect of 7-Met in OXZ-induced mice model, the pathway and the functional enrichment analysis of differentially expressed chemokines mRNAs in OXZ-induced model were performed, and the major protein–protein interaction (PPI) network of OXZ-induced differentially expressed chemokines mRNAs were mainly enriched in the IL-17 signaling pathway (Fig. 6a, Supplementary Tables 3–5). To confirm the validity of the PPI network data, RT-qPCR and Western blotting were undertaken for putative chemokines genes (Cxcl1, Cxcl2, and Cxcl3). The results were consistent with PPI network data (Fig. 6b). RT-qPCR data also showed that IL-17A decreased remarkably in the 7-Met group (Fig. 6c). The Th17 cells constitute a unique subset of CD4+ T cells and are the major source of IL-17^42,43^. Studies have revealed that the IL-17 and Th17 cells are involved in the pathogenesis of AD. To investigate how 7-Met regulates Th17 cells, the IL-17+ Th17 cells were examined by Immunohistochemistry staining and the transcription factors (e.g., STAT3) were determined by Western blotting. IL-17+ Th17 cells were decreased remarkably versus OXZ model (Fig. 6d,e). Consistently, STAT3 and phospho-STAT3, specific Th17 transcription factors, were found to be downregulated in 7-Met-treated group (Fig. 6f).

We further investigated the MAPK-AP1 signaling pathway by Western blotting, as IL-17 leads to the activation of MAPK signaling pathway^14^. We found that 7-Met administration effectively reversed activation of Ras, c-Raf and MAPK proteins (including ERK, JNK and p38 MAPK) in mice ear tissue and dorsal skin, as indicated by their protein expression and phosphorylation (Fig. 6f,g). Additionally, 7-Met suppressed the AP-1 transcriptional activity in AD mice by decreasing the expression of AP-1 components c-Fos and c-Jun (Fig. 6h).

## Discussion

In the current study, we have investigated the anti-AD activity of 7-Met in BALB/c mice with FITC or OXZ-induced AD. We found that topical treatment of 7-Met ameliorated FITC or OXZ-induced AD-like skin lesions and improved skin erosion severity, ear swelling and epidermal thickness in FITC or OXZ-treated BALB/c mice (Figs. 1, 2). Administration of 7-Met decreased the serum levels of IFN-γ, IL-4 and IL-17A compared to those in the model groups. Our findings revealed that 7-Met modulated the AD-related immune imbalance by regulating the responses of AD-related T cell subsets, including Th17, as well as the Th1/Th2 balance. 7-Met was validated to attenuate neutrophilic inflammation by inhibiting the MAPK-AP1 signaling pathway. Moreover, as illustrated by the spleen index, 7-Met regulated the overactivated immune response in AD mice with much fewer side effects compared to dexamethasone, a widely used anti-dermatitis drug. Hence, our study suggests that 7-Met could be a promising potential therapeutic agent against AD.

Modulating T cell-elicited immune responses is one of the promising therapeutic approaches for AD. Fan et al. confirmed that rosae multiflorae fructus extract and its four active components alleviated atopic dermatitis via regulation of Th1/Th2 imbalance in BALB/c rhinitis mice^44^. Moreover, Guttman-Yassky et al. confirmed that dupilumab, a monoclonal antibody that specifically targets IL-4Rα, thereby blocking the Th17/Th22 pathway, is highly efficacious for controlling skin disease in moderate-to-severe AD patients^45^. In this study, two AD mice models were established to evaluate the anti-AD activity of 7-Met and its potential mechanisms. However, limited aspects of CD4+ naive T cell differentiation were reflected, and there remains a considerable translational gap between AD mice models and human AD^24,25,30^. As expected, FITC and OXZ induced AD in different ways as FITC changed Th1/Th2 balance while OXZ increased Th17 cells sub-sets. 7-Met was shown to function differently in the two models as it restored the Th1/Th2 balance in FITC-induced mice model whereas it modulated the responses of the Th17 cells subsets in OXZ-induced mice model.

High-throughput assessment of gene expression in patient tissues using microarray technology or RNA-Seq took a center stage in clinical research of skin diseases during the last decades. Consequently, in order to clarify the potential mechanisms, high throughput transcriptome sequencing was performed on the 7-Met treated AD mice induced by either FITC or OXZ. KEGG and GO enrichment showed that the primary pathway of genes regulated by 7-Met was associated with chemokine signaling pathways, cytokine-cytokine receptor interaction and the chemotaxis of multiple categories of cells. These clues from the RNA-Seq results were confirmed by our RT-qPCR and Western blotting analysis. Similarly, Joanna et al.^46^ assessed the serum levels of Th1- and Th2-derived chemokines in AD patients and concluded that chemokine imbalance was involved in AD pathogenesis. Recently, Cole et al.^47^ adopted RNA-seq to characterize the increases in expression in the Th1 and Th2 chemokine (e.g., Cxcl10 and Ccl18) in nonregional AD skin.

However, not all biological functions are reliably reflected by the transcriptome, and more information may be detected in the proteome or phosphoproteomics. Single-cell RNA sequencing (scRNA-seq) technology enables the identification of cellular heterogeneity in far greater detail than traditional methods by measuring transcriptomes at the single-cell level. There is no doubt that this technology can accurately reflect the subset change, differentiation and phenotypic transformation of immune cells during the pathogenesis of atopic dermatitis in mice, which is meaningful for drug development of atopic dermatitis.

It is widely acknowledged that atopic dermatitis, as a heterogeneous disease, has symptoms and pathologic processes partially similar to psoriasis^48^. These two diseases have homogeneous pathologic course resulted from multiple factors including, for example, chronic inflammatory response, abnormal function of dendritic cell^19^, infiltration of neutrophil^49^, abnormal increase in keratinocytes^50^, and a variety of other mechanisms^51^. Regardless of some heterogeneity between these two diseases, a recent study revealed that Asian atopic dermatitis phenotype combines the characteristics of atopic dermatitis and psoriasis, accompanied by marked Th17 polarization^43^.

In this study, FITC and OXZ were employed to simulate two models of atopic dermatitis with slightly different mechanisms. It was demonstrated that OXZ-induced atopic dermatitis induced the polarization of Th17 cell subsets, mechanistically similar to psoriasis. According to previous work, keratinocyte and dendritic cell function in the psoriasis course are related to TSLP^52^, which abnormally increased in FITC-induced atopic dermatitis mice. According to our observations, 7-Met can significantly inhibit chronic inflammation of mouse skin, reduce infiltration of neutrophils, alleviate the abnormal increase of keratinocytes, alleviate abnormal differentiation of Th17 cell subsets in OXZ-induced mouse models, and effectively attenuate FITC-induced excessive secretion of TSLP. All these biological regulatory dominos suggest that 7-Met may be adopted as a potential drug for atopic dermatitis and presents considerable clinical significance and high research value in the psoriasis drug development^53,54^.

## Conclusion

**Figure 7:**
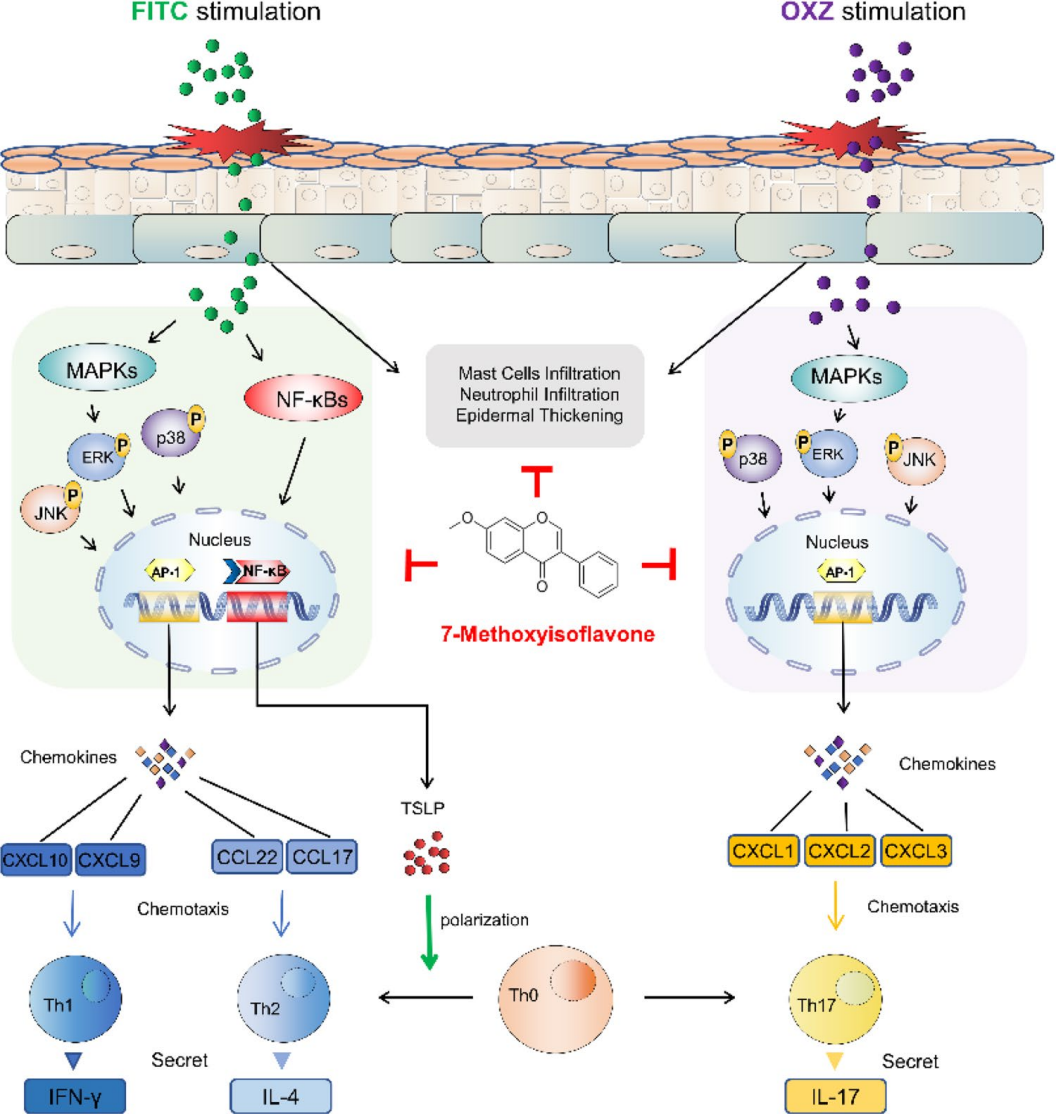
7-Methoxyisoflavone ameliorated AD symptoms in two different models. Topical 7-Methoxysioflavone modulates Th1/Th2 cytokine levels, attenuates mast cell infiltration, inhibits MAPKs and NF-κB signaling, reducing TSLP production and ameliorating inflammation in FITC (green)-induced atopic dermatitis mice. While this anti-inflammatory effect is achieved mainly through the inhibition of Th17-related chemokines production mediated by MAPKs-AP1 signaling in OXZ (purple)-induced atopic dermatitis mice (This figure was drawn by Hao Dong).

In this work, we have evaluated the therapeutic effects of 7-Met on mice with atopic dermatitis induced by FITC or OXZ. We found that topical treatment of 7-Met ameliorated FITC or OXZ-induced AD-like skin lesions and improved skin erosion severity, ear swelling and epidermal thickness in FITC or OXZ-treated BALB/c mice. RNA-seq was performed followed by KEGG and GO analysis to study global changes in gene expression in two different AD mice models for the first time. Our study showed that 7-Methoxyisoflavone alleviated atopic dermatitis by regulating Th1/Th2 balance in FITC-induced AD model and reduced Th17 cell subset in OXZ-induced AD model, suggesting that 7-Methoxyisoflavone could be a promising potential therapeutic agent against AD. (Fig. 7).

## Materials and methods

### Materials

The reagents used in this study were purchased as follows. Fluorescein isothiocyanate (FITC) and oxazolone (OXZ) were purchased from Sigma (Sigma‐Aldrich China, Shanghai, China). Olive oil was purchased from Shanghai Lingfeng Chemical Reagent Co. Ltd (Shanghai, China). Acetone was purchased from Nanjing Chemical Reagent Co. Ltd (Nanjing, China). Dibutyl phthalate (DBP) was obtained from Aladdin (Shanghai, China), and Compound Dexamethasone Cream was a product from Guangzhou Baiyunshan Pharmaceutical Co Ltd (Guangzhou, China). 7-Methoxyisoflavone (7-Met) was purchased from MedChemExpress (Shanghai, China).

### Animals

BALB/c female mice (18–20 g, 6 weeks of age) were purchased from College of Veterinary Medicine Yangzhou University (institute of comparative medicine) (Yangzhou, China). Mice were housed in a specific pathogen-free conditions room with a 12 h light/dark cycle at 25 ± 2 °C and 50–60% humidity. Mice were given access to a standard laboratory diet and water ad libitum. All mouse experiments were performed following the guidelines of the Laboratory Animal Center of China Pharmaceutical University, and approved by the animal experiment committee of China Pharmaceutical University. All animal experiments were carried out in accordance with ARRIVE guidelines (https://arriveguidelines.org/).

### Induction of atopic dermatitis and treatment of drug in mice

After one week of acclimation, mice were randomly divided into 3 groups of 8 mice each: (1) control group without FITC/OXZ application; (2) model group with 0.5% FITC or OXZ application; (3) 2.5% 7-Met-treated group with 0.5% FITC/OXZ application. Mice were anesthetized with 2% isoflurane and the dorsal hair was shaved with an area of 3.5 cm × 3.5 cm on day 0. 0.5% FITC or 0.5% OXZ dissolved in 40 μL vehicle (acetone: DBP = 1:1 or ace-tone: olive oil = 4:1) was used as sensitizer on the dorsal hairless area on days 1 and 2. The control group was administrated with 40 μL vehicle. On the 6th day, 40 μL 0.5% FITC or 0.5% OXZ solution was used to stimulate both dorsal skin and the right ear. The control group was challenged with 40 μL vehicle. All mice were applied with 20 μL vehicle on the left ear. Mice were sacrificed on Day 7, and blood was collected by extirpating eyeballs and stored at − 80 °C for further analysis. The dorsal skin and ear tissues of mice were excised and subjected to histological examination. 2.5% 7-Met was dissolved in emulsions. The same volume of vehicle was applied to the control and model groups. Seven days after the first FITC/OXZ application, 7-Met or volume were applied to the dorsal skin and ears daily for 7 days.

### Preparation of emulsions

Stearic acid (10%), glyceryl monostearate (2%), beeswax (2%) and palm oil (6%) were added into a beaker, and mixed as the oil phase. Distilled water (71.94%), 1,3-Butanediol (6%), ethanol (2%), carbomer (0.03%) and triethanolamine (0.02%) were added into the other beaker, and mixed as the aqueous phase. Both the oil phase and the aqueous phase were heated to 80 ℃ using water bath cauldron, and then mixed using a mechanical stirrer until the mixture becomes homogenous. The emulsions were kept in air tight containers.

### Evaluation of ear swelling and spleen index

Ear thickness was measured in the central portion of each ear lobe before mice were sacrificed using micrometer caliper (Mitutoyo Corporation, Kanagawa, Japan). The difference of right ear thickness and left ear thickness was regarded as ear thickness. Weights of spleen was measured with an electronic balance (ME55, METTLER TOLEDO, China) and the ratio of spleen weight to body weight was regarded as spleen index.

### Histological and immunohistochemical analysis

Biopsied tissue was fixed in 4% formalin, embedded in paraffin, sectioned at 4 μm, and stained with hematoxylin and eosin (H&E) and toluidine blue (TB). Infiltrated lymphocytes, thickening of the epidermis, and fibrosis in the dermis were observed using H&E-stained tissue sample under a magnification of × 400. Thickness was measured in five randomly selected fields from each sample. Mast cell infiltration was measured by counting the number of mast cell in four sites chosen at random in biopsies stained with toluidine blue at a magnification of × 400. For immunohistochemical staining, sections were blocked with 10% BSA for 2 h, followed by overnight incubation with a primary antibody against IL-17A (Abcam), CD11b (Service Bio, Wuhan) and CK14 (Service Bio, Wuhan) at 4 °C. Subsequently, sections were washed and incubated with horseradish peroxidase-conjugated secondary antibodies for 1 h at room temperature. All stained skin sections were observed using an inverted microscope (IX73, Olympus, Japan).

### Measurement of IgE, IL-4, IL-13 and IFN-γ levels

The serum levels of IgE, IL-4 and IFN-γ were measured using mouse enzyme-linked immunosorbent assay (ELISA) kit (Senbeijia, Nanjing) according to the protocol provided by the manufacturer.

The IL-13 levels in mouse dorsal skin and ears homogenate were measured using mouse enzyme-linked immunosorbent assay (ELISA) kit (Elab Science, Wuhan) according to the protocol provided by the manufacturer.

### Western blotting

Protein lysates were prepared using RIPA Lysis Buffer (Beyotime Biotechnology, China) containing protease inhibitors (KGP602). Proteins were separated by 10% SDS-PAGE and transferred onto PVDF membrane (Millipore, China). Membranes were blocked with 5% nonfat milk or 5% bovine serum albumin (Sigma-Aldrich China, Shanghai, China) and then incubated at 4 °C for 24 h with specific primary antibodies against Ras(27H5) (Cell Signaling Technology, Inc, China, #3339); c-Raf(D4B3J) (CST, #53745); an-ti-raf1(phospho-S259) (Abcam, MA, USA, #173539); p38 MAPK(D13E1, #8690) (CST); phospho-p38 MAPK(Thr180/Tyr182) (D3F9) (CST, #4511); SAPK/JNK (CST, #9252); phospho-SAPK/JNK (Thr183/Tyr185) (CST, #9255); p44/42MAP Kinase (CST, #4696); phos-pho-p44/42MAPK(ERK1/2) (Thr202/Tyr204) (E10) (CST, #9106); anti-MEK1 + MEK2(Abcam, #178876); phospho-MEK(Ser217/221) (41G9) (CST, #9154); c-Fos(9F6) (CST, #2250); c-Jun(60A8) (CST, #9165); IκBα(L35A5) (CST, #4814); phospho-IκBα(Ser32/36) (CST, #9246); STAT3(124H6) (CST, #9139); phospho-STAT3(Tyr705) (CST, #4113); anti-COX2(Abcam, #52237); β-actin(Abcam); GAPDH(Abcam); PCNA(D3H8P) (CST, #13110); anti-Cxcl1/GRO alpha(Abcam, #86436); anti-Cxcl2(Abcam, #25130); anti-Cxcl3/GRO gamma(Abcam, #220431). Ccl, C–C motif chemokine ligand; Cxcl, C–X–C motif chemokine ligand. Membranes were then incubated with secondary antibodies for 2 h at room temperature. Membranes were treated with the enhanced chemiluminescence (ECL) (KeyGen, China) detection reagent (KeyGen, China) and visualized by GelDoc XR System (Bio-Rad, USA). All the blot images in each panel were exposed using the same parameters and taken from different parts of a same gel. In order to avoid the interference of non-specific binding of antibodies to chemiluminescence imaging. Under the premise of ensuring credibility, some WB images are cropped strips, all images have been provided in the Supplementary Document.

### Real-time quantitative PCR

After rapid dissection of ear, tissue was preserved in RNA-later (Sigma-Aldrich, St. Louis, MO, USA) at 4 °C before storing at − 80 °C until use. Total RNA was obtained from ear tissue using the FastPure^®^ Cell/Tissue Total RNA Isolation Kit (RC101, Vazyme, Nanjing, China) according to the manufacturer’s instructions. After the RNA extraction, RNA was quantified using a NanoDrop ND-2000 spectrophotometer (Thermo Fisher Scientific, Wilmington, DE, USA). The first-strand complementary DNA (cDNA) was synthesized using HiScript III 1st Strand cDNA Synthesis Kit (Vazyme, Nanjing, China). Quantitative real-time polymerase chain reaction (qRT-PCR) was executed with synthesized cDNA templates, forward and reverse primers (GenScript Biological Technology, co., Ltd.) and ChamQ SYBR Color qPCR Master Mix (q421, Vazyme, Nanjing, China) into the Bio-Rad CFX Connect Real-Time PCR Detection System (CFX96, Bio-Rad, Hercules, California, USA). The primers were designed based on the mRNA sequences obtained from the NCBI database, and the primer sequences are shown in Supplementary Table 1. Accumulated PCR products were detected directly by monitoring the increase in the reporter dye (SYBR Green). The expression levels of cytokines in the exposed cells were compared to the expression levels in control cells at each collection time point using the comparative cycle threshold (Ct) method. The mRNA expression level of each gene was calculated from the cycle threshold (Ct) value using the ΔΔCt method and normalized to GAPDH.

### Preprocessing of high-throughput transcriptome sequencing data

The sequencing data was filtered with SOAPnuke (v1.5.2)^55^ by (1) Removing reads containing sequencing adapter; (2) removing reads whose low-quality base ratio (base quality less than or equal to 5) is more than 20%; (3) removing reads whose unknown base (‘N’ base) ratio is more than 5%, afterwards clean reads were obtained and stored in FASTQ format. The clean reads were mapped to the reference genome using HISAT2 (v2.0.4)^56^. Bowtie2 (v2.2.5)^57^ was applied to align the clean reads to the reference coding gene set, then expression level of gene was calculated by RSEM (v1.2.12)^58^. The heatmap was drawn by pheatmap (v1.0.8)^59^ according to the gene expression in different samples. Essentially, differential expression analysis was performed using the DESeq2(v1.4.5) with Q value ≤ 0.05. To take insight to the change of phenotype, Gene Ontology (GO) (http://www.geneontology.org/) and Kyoto Encyclopedia of Genes and Genomes (KEGG) (https://www.kegg.jp/) enrichment analysis of annotated different expressed gene was performed by Phyper (https://en.wikipedia.org/wiki/Hypergeometric_distribution) based on Hypergeometric test. The significant levels of terms and pathways were corrected by Q value with a rigorous threshold (Q value ≤ 0.05) by Bonferroni (Supplementary Table 2).

### Statistical analysis

Quantitative data are presented as mean ± sem (standard error). The statistical significance of differences between groups was examined by Ordinary one-way ANOVA (for three groups) using GraphPad PRISM 8.0 software (GraphPad Software, La Jolla, CA, USA). A p-value less than 0.05 was considered significant.

### Institutional review board statement

The study was approved by the Institutional Review Board (or Ethics Committee) of Ethics Committee of China Pharmaceutical University (protocol code 2021-01-016). All animal experiments were carried out in accordance with ARRIVE guidelines (https://arriveguidelines.org/).

## Supplementary Information

Supplementary Tables.

Supplementary Information.

## Data Availability

Accession to cite for these SRA data: PRJNA688463; Temporary Submission ID: SUB8807646; Release date: 2021-06-30; Accession to cite for these SRA data: PRJNA688640; Temporary Submission ID: SUB8811554; Release date: 2021-06-30.
