# Supplementary material for: 7-Methoxyisoflavone ameliorates atopic dermatitis symptoms by regulating multiple signaling pathways and reducing chemokine production
Source: Sci Rep. 2022 May 24;12:8760. doi: 10.1038/s41598-022-12695-3 (PMC9130209; doi:10.1038/s41598-022-12695-3)
Supplement: Supplementary file 1 — Supplementary Tables. [file 41598_2022_12695_MOESM1_ESM.docx]

Supplementary Table 1. qRT-PCR primers used in this study

| **Gene Name** | **Forward** | **Reverse** |
| --- | --- | --- |
| IL-17A | CCTGGCTTTTGTCTCCCCTG | TTCCCTCCGCATTGACACAG |
| Ccl17 | ATAGGAGGGGACAGGAAGCG | GCCAGGAGCAGCATCTGAAG |
| Ccl22 | GCAGGTCTGGGTGAAGAAGC | GGTGAGTAAAGGTGGCGTCG |
| Cxcl1 | GATTCACCTCAAGAACATCCAG | TGGGGACACCTTTTAGCATC |
| Cxcl2 | CGCTGTCAATGCCTGAAGAC | ACACTCAAGCTCTGGATGTTCTTG |
| Cxcl3 | GAAGATTACTGAAGAGCGGCAAGTC | AATGCAGGTCCTTCATCATGGT |
| Cxcl9 | TTTCATCACGCCCTTGAGCC | GTCTTCGGAGGCTGCCAAAT |
| Cxcl10 | CCTTTTGCCCCAGGGTCTTT | CTGTCCATCCATCGCAGCAC |
| GAPDH | TTTGCCGTGAGTGGAGTCAT | GGTCCAGGGGTCTTACTC |

Supplementary Table 2. Software or programming packages used in this study

| **Software** | **Source** |
| --- | --- |
| SOAPnuke (v1.5.2) | https://github.com/BGI-flexlab/SOAPnuke |
| Bowtie2 (v2.2.5) | http://bowtiebio.sourceforge.net/%20Bowtie2%20/index.shtml |
| RSEM (v1.2.12) | https://github.com/deweylab/RSEM |
| Pheatmap (1.0.8) | https://cran.r-project.org/web/packages/pheatmap/index.html |
| DESeq2 (v1.4.5) | http://www.bioconductor.org/packages/release/bioc/html/DESeq2.html |
| HISAT2 (v2.0.4) | http://www.ccb.jhu.edu/software/hisat/index.shtml |
| Cytoscape (v3.8.2) | https://cytoscape.org/download.html |

Supplementary Table 3. KEGG Enrichment of PPI network in OXZ-induced AD Models

| **#Term ID** | **Term Description** | **Strength** | **False Discovery Rate** |
| --- | --- | --- | --- |
| mmu04062 | Chemokine signaling pathway | 2.02 | 1.78E-67 |
| mmu04060 | Cytokine-cytokine receptor interaction | 1.85 | 3.45E-60 |
| mmu04657 | IL-17 signaling pathway | 1.78 | 7.62E-16 |
| mmu04668 | TNF signaling pathway | 1.57 | 4.97E-10 |
| mmu05323 | Rheumatoid arthritis | 1.64 | 2.46E-09 |
| mmu05132 | Salmonella infection | 1.51 | 3.10E-06 |
| mmu04621 | NOD-like receptor signaling pathway | 1.26 | 4.36E-06 |
| mmu04620 | Toll-like receptor signaling pathway | 1.41 | 6.35E-06 |
| mmu04672 | Intestinal immune network for IgA production | 1.7 | 6.35E-06 |
| mmu05142 | Chagas disease (American trypanosomiasis) | 1.3 | 0.00016 |
| mmu05134 | Legionellosis | 1.42 | 0.00061 |
| mmu04623 | Cytosolic DNA-sensing pathway | 1.39 | 0.00068 |
| mmu05164 | Influenza A | 1.08 | 0.00079 |
| mmu04064 | NF-kappa B signaling pathway | 1.21 | 0.0019 |
| mmu05144 | Malaria | 1.35 | 0.0077 |
| mmu05167 | Kaposi's sarcoma-associated herpesvirus infection | 0.87 | 0.0143 |
| mmu05168 | Herpes simplex infection | 0.87 | 0.0143 |
| mmu04144 | Endocytosis | 0.77 | 0.0244 |
| mmu04933 | AGE-RAGE signaling pathway in diabetic complications | 1 | 0.0269 |
| mmu04670 | Leukocyte trans-endothelial migration | 0.94 | 0.033 |
| mmu05418 | Fluid shear stress and atherosclerosis | 0.85 | 0.0449 |
| mmu04072 | Phospholipase D signaling pathway | 0.84 | 0.0457 |

Supplementary Table 4. GO Functions Enrichment of PPI network in OXZ-induced AD Models

| **#Term ID** | **Term Description** | **Strength** | **False Discovery Rate** |
| --- | --- | --- | --- |
| GO:0008009 | chemokine activity | 2.59 | 3.57E-71 |
| GO:0048020 | CCR chemokine receptor binding | 2.41 | 4.30E-36 |
| GO:0045236 | CXCR chemokine receptor binding | 2.57 | 7.07E-19 |
| GO:0005515 | protein binding | 0.48 | 3.76E-15 |
| GO:0016494 | C-X-C chemokine receptor activity | 2.57 | 1.31E-12 |
| GO:0048248 | CXCR3 chemokine receptor binding | 2.6 | 1.40E-08 |
| GO:0019956 | chemokine binding | 2.02 | 2.19E-08 |
| GO:0019958 | C-X-C chemokine binding | 2.46 | 3.25E-08 |
| GO:0005488 | binding | 0.27 | 3.82E-08 |
| GO:0031726 | CCR1 chemokine receptor binding | 2.57 | 1.60E-06 |
| GO:0031735 | CCR10 chemokine receptor binding | 2.57 | 1.60E-06 |
| GO:0008201 | heparin binding | 1.34 | 2.15E-06 |
| GO:0031727 | CCR2 chemokine receptor binding | 2.48 | 2.15E-06 |
| GO:0004918 | interleukin-8 receptor activity | 2.7 | 0.0001 |
| GO:0031728 | CCR3 chemokine receptor binding | 2.7 | 0.0001 |
| GO:0042056 | chemoattractant activity | 1.71 | 0.00014 |
| GO:0019959 | interleukin-8 binding | 2.52 | 0.00015 |
| GO:0031732 | CCR7 chemokine receptor binding | 2.22 | 0.00042 |
| GO:0031730 | CCR5 chemokine receptor binding | 2.16 | 0.00052 |
| GO:0016004 | phospholipase activator activity | 1.96 | 0.0011 |

Supplementary Table 5. GO Process Enrichment of PPI network in OXZ-induced AD Models

| **#Term ID** | **Term Description** | **Strength** | **False Discovery Rate** |
| --- | --- | --- | --- |
| GO:0070098 | chemokine-mediated signaling pathway | 2.39 | 1.14E-67 |
| GO:0060326 | cell chemotaxis | 2 | 1.92E-61 |
| GO:0006935 | chemotaxis | 1.64 | 1.08E-55 |
| GO:0030595 | leukocyte chemotaxis | 2.12 | 2.15E-54 |
| GO:0048247 | lymphocyte chemotaxis | 2.46 | 7.74E-47 |
| GO:0002685 | regulation of leukocyte migration | 1.87 | 5.35E-44 |
| GO:0030593 | neutrophil chemotaxis | 2.3 | 5.95E-44 |
| GO:0016477 | cell migration | 1.38 | 5.57E-43 |
| GO:0097529 | myeloid leukocyte migration | 2.09 | 7.00E-42 |
| GO:0002687 | positive regulation of leukocyte migration | 1.94 | 1.26E-40 |
| GO:0010469 | regulation of signaling receptor activity | 1.46 | 1.14E-39 |
| GO:0034097 | response to cytokine | 1.33 | 1.43E-38 |
| GO:0002688 | regulation of leukocyte chemotaxis | 2 | 3.24E-38 |
| GO:0002690 | positive regulation of leukocyte chemotaxis | 2.07 | 6.53E-38 |
| GO:0050920 | regulation of chemotaxis | 1.81 | 1.27E-37 |
| GO:0050921 | positive regulation of chemotaxis | 1.91 | 2.01E-36 |
| GO:0006955 | immune response | 1.26 | 8.92E-35 |
| GO:0006954 | inflammatory response | 1.49 | 1.78E-34 |
| GO:0040017 | positive regulation of locomotion | 1.42 | 4.42E-34 |
| GO:0030335 | positive regulation of cell migration | 1.45 | 2.23E-33 |
