# Supplementary material for: 7-Methoxyisoflavone ameliorates atopic dermatitis symptoms by regulating multiple signaling pathways and reducing chemokine production
Source: Sci Rep. 2022 May 24;12:8760. doi: 10.1038/s41598-022-12695-3 (PMC9130209; doi:10.1038/s41598-022-12695-3)

Western Blotting Original Images

Figure5h

RAS

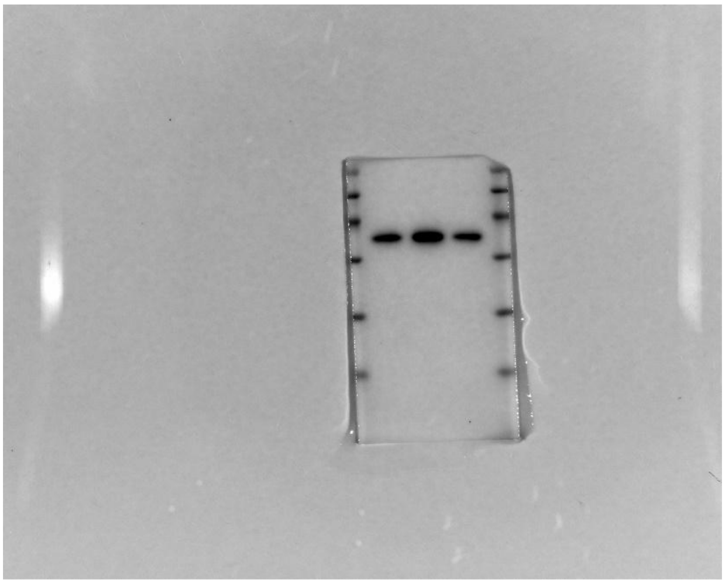

ERK

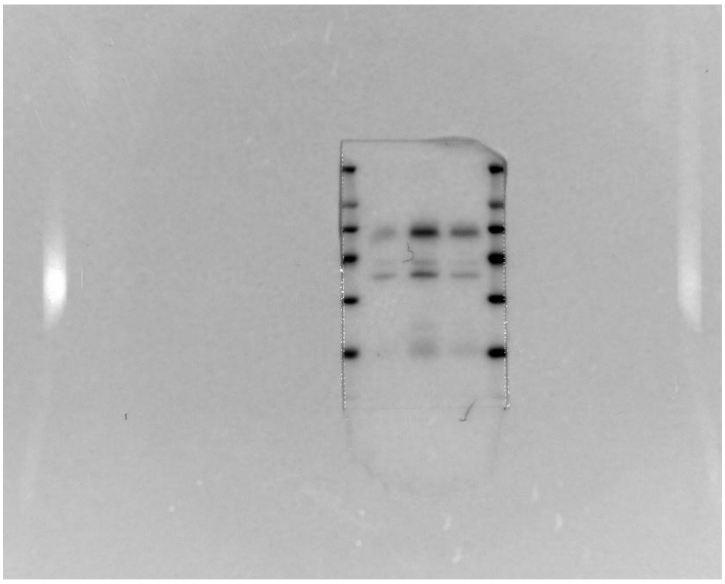

p-ERK

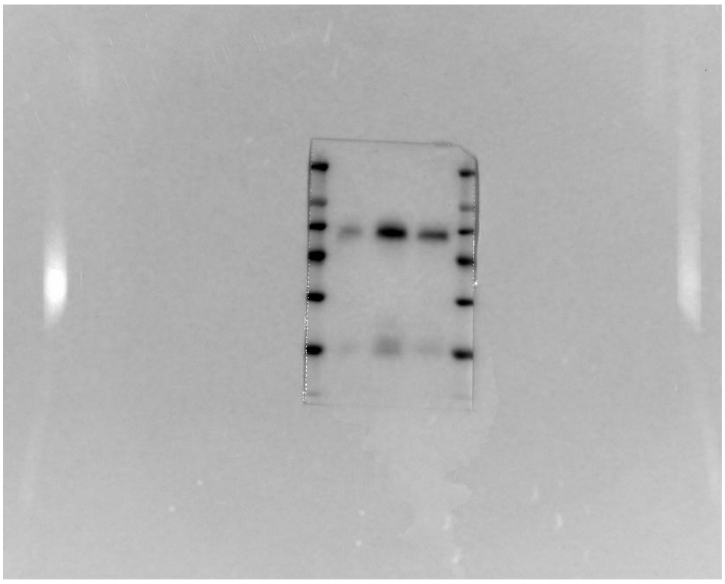

JNK

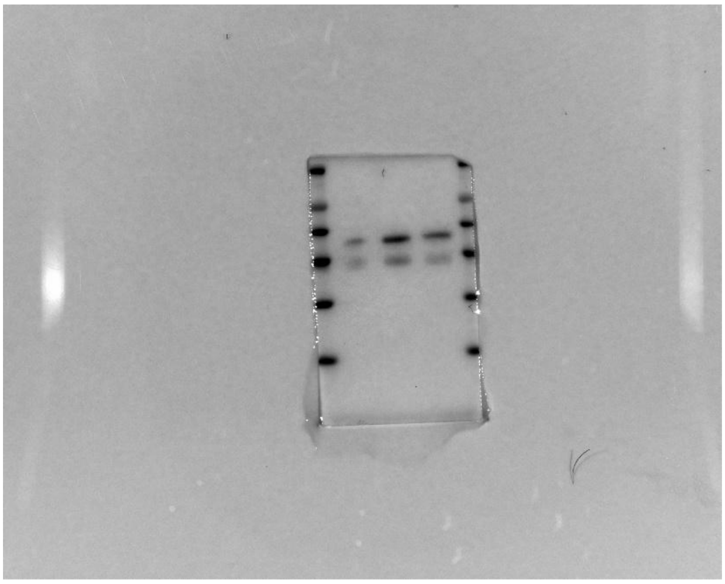

p-JNK

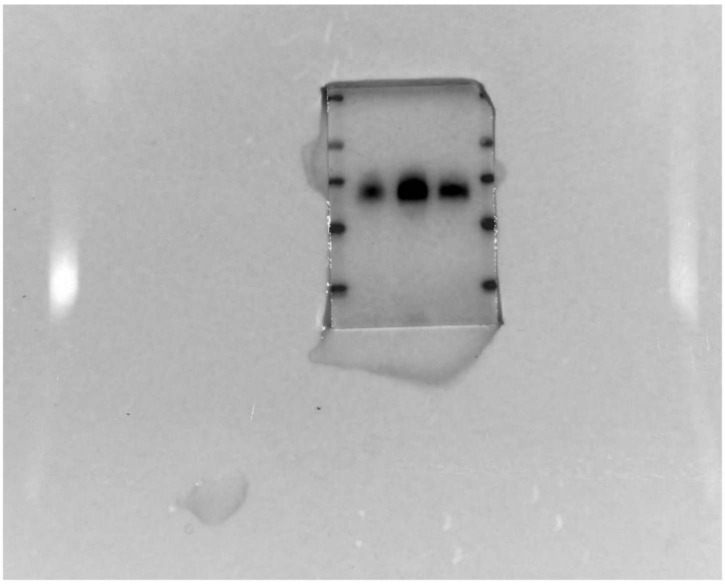

P38

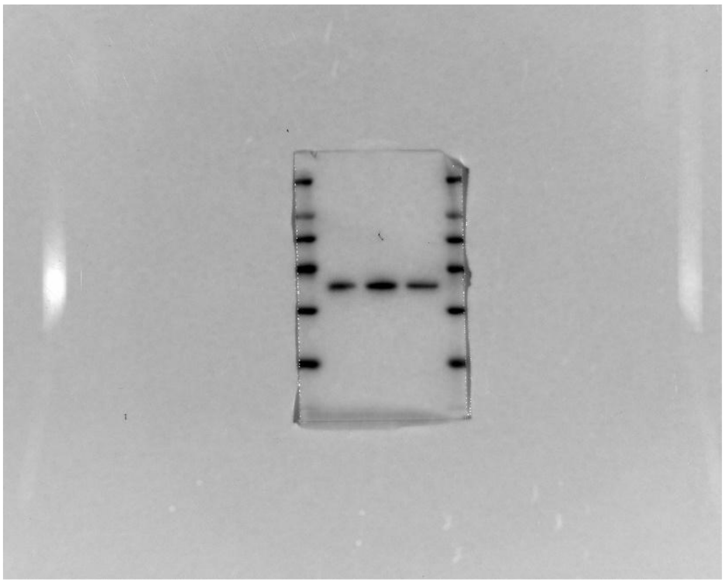

p-p38

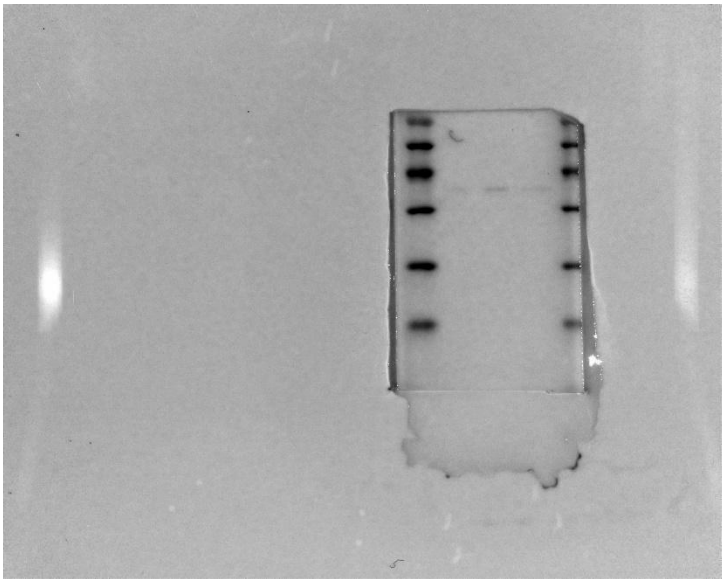

Stat3

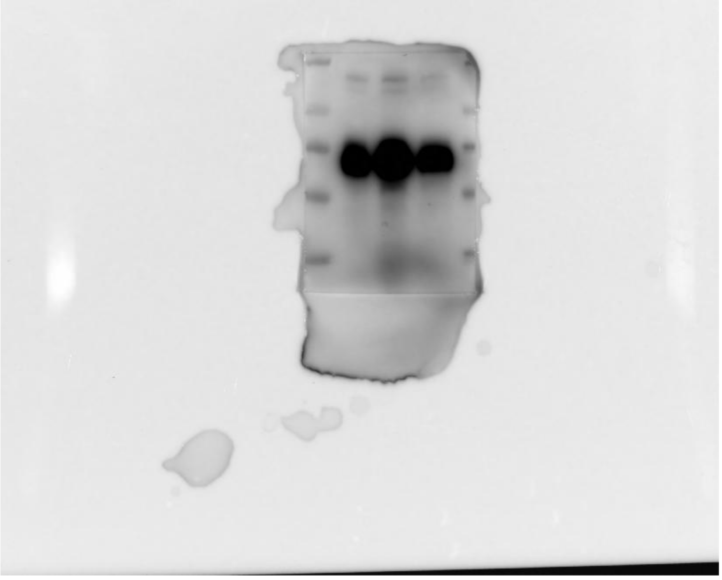

p-stat3

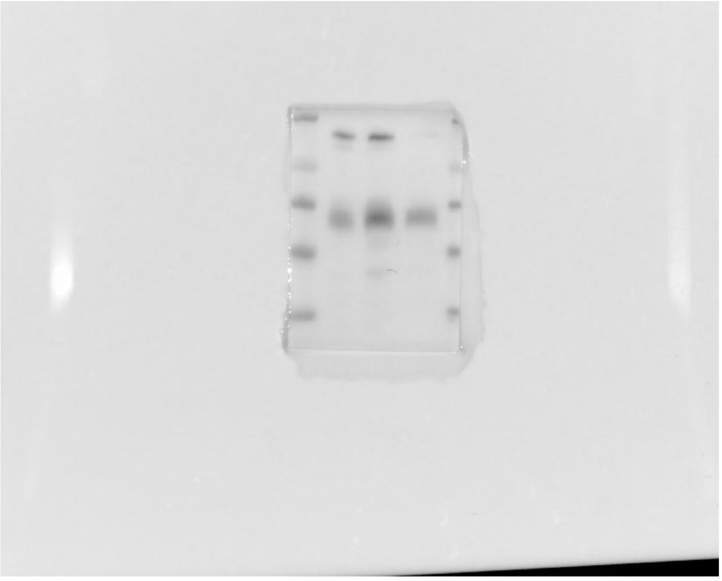

TSLP

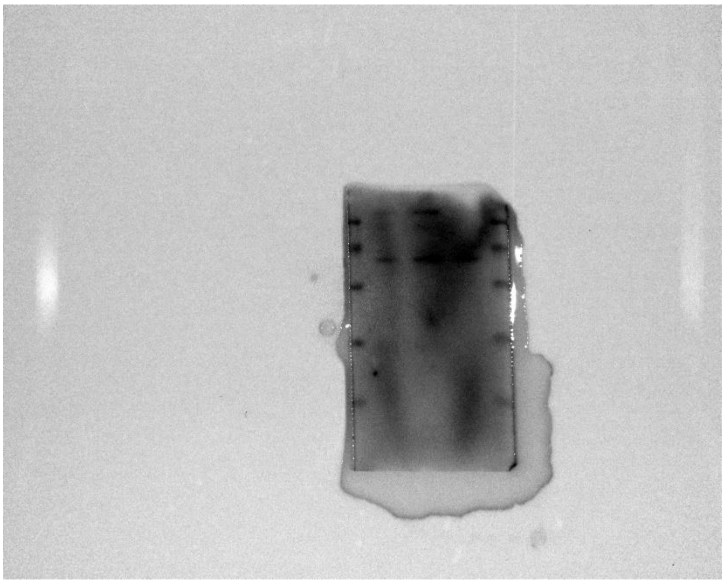

GAPDH

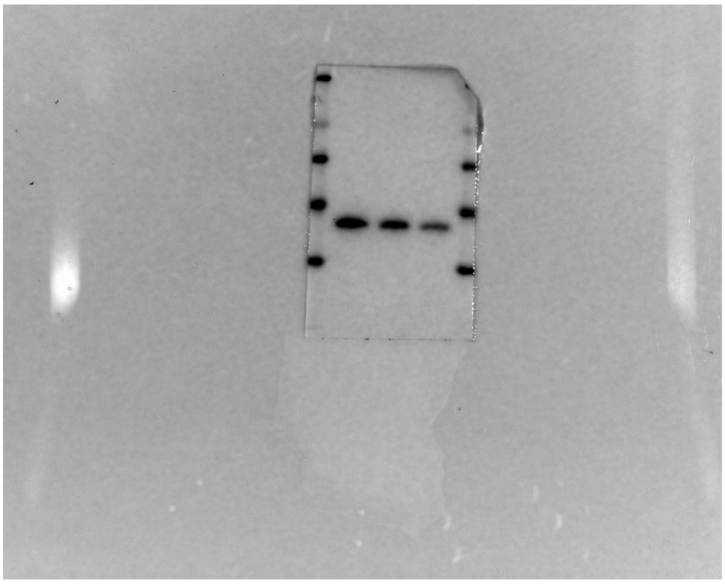

Figure5i

ERK

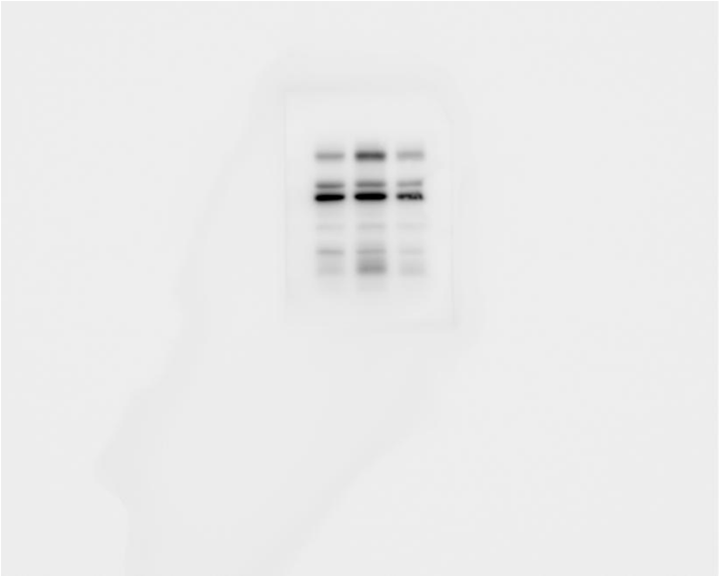

p-ERK

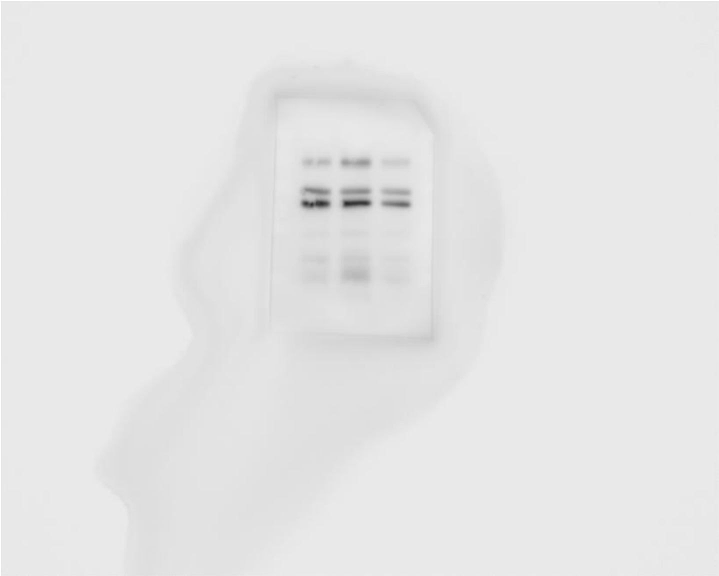

JNK

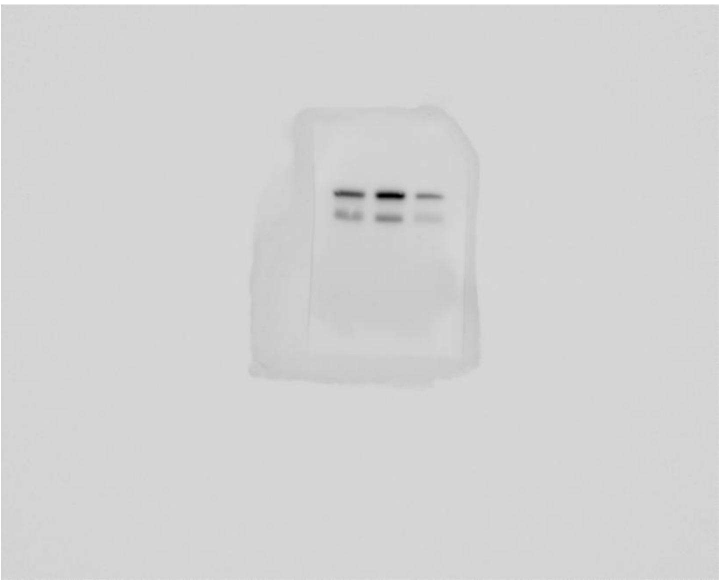

p-JNK

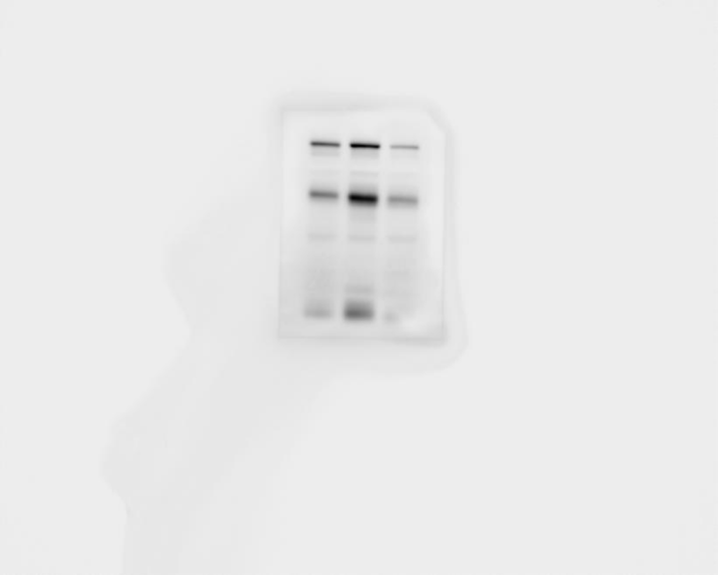

p38

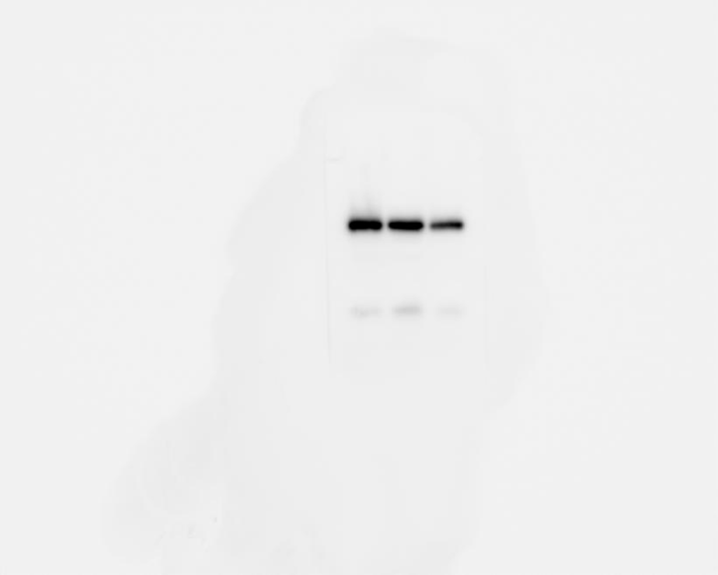

p-p38

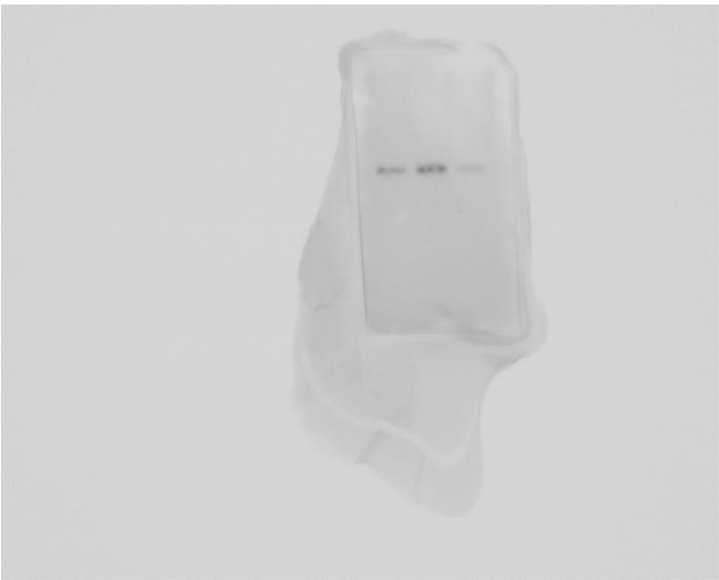

GAPDH

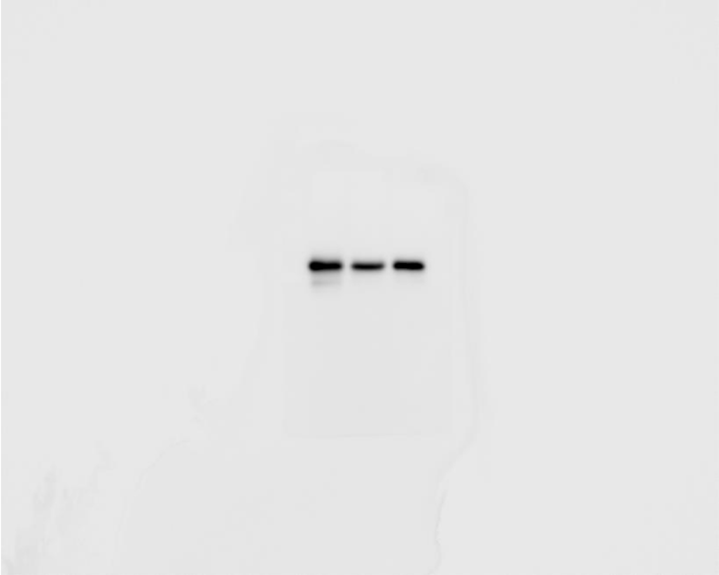

Figure5j

c-Fos

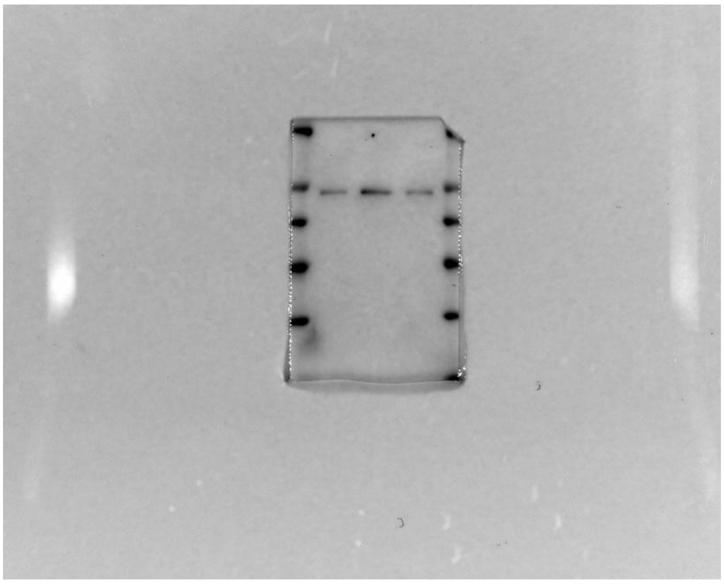

c-Jun

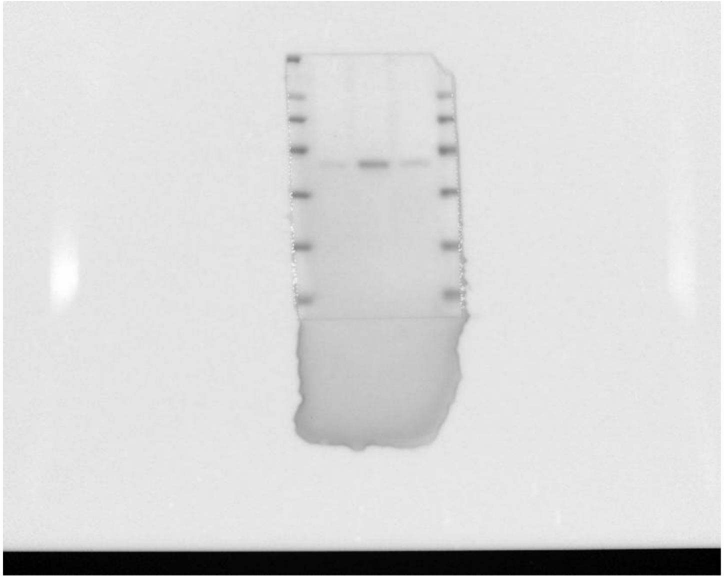

p-IkBa

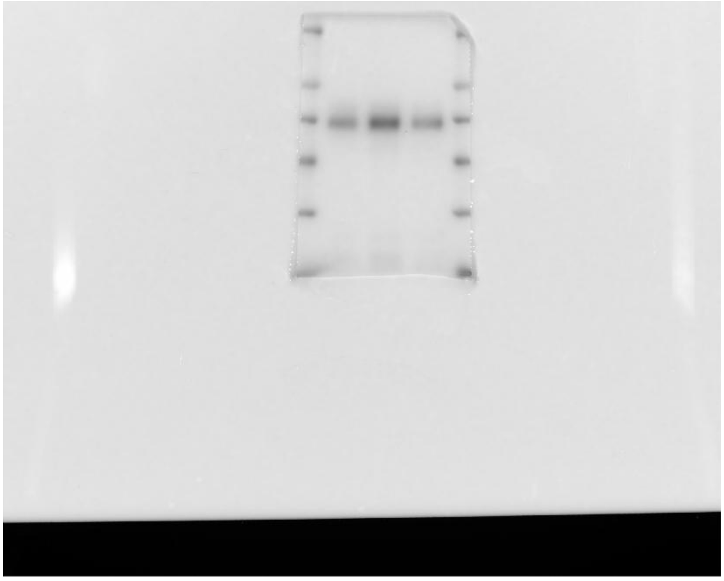

p-p65

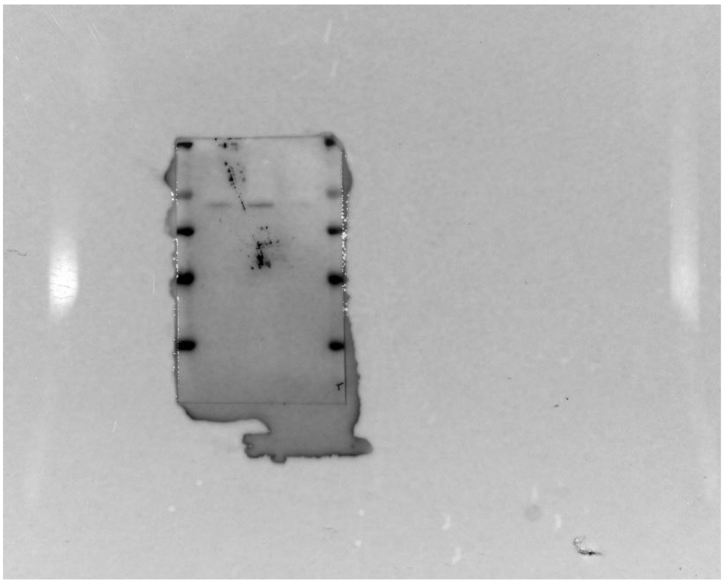

PCNA

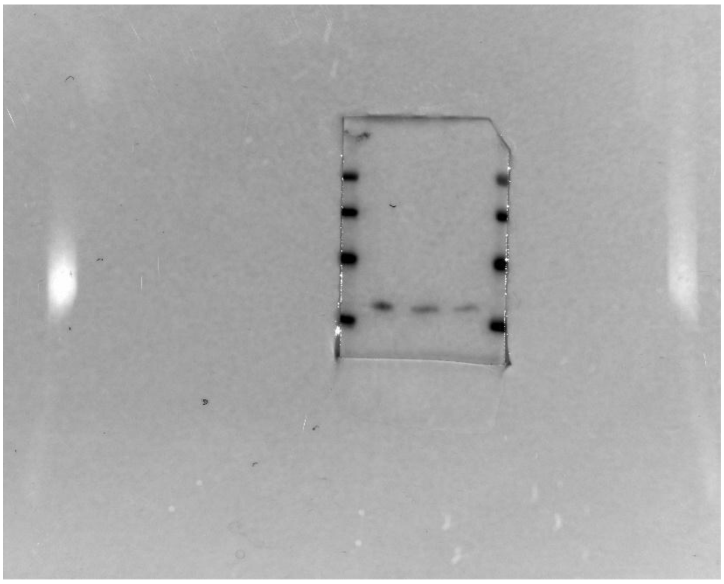

Figure6f

c-raf

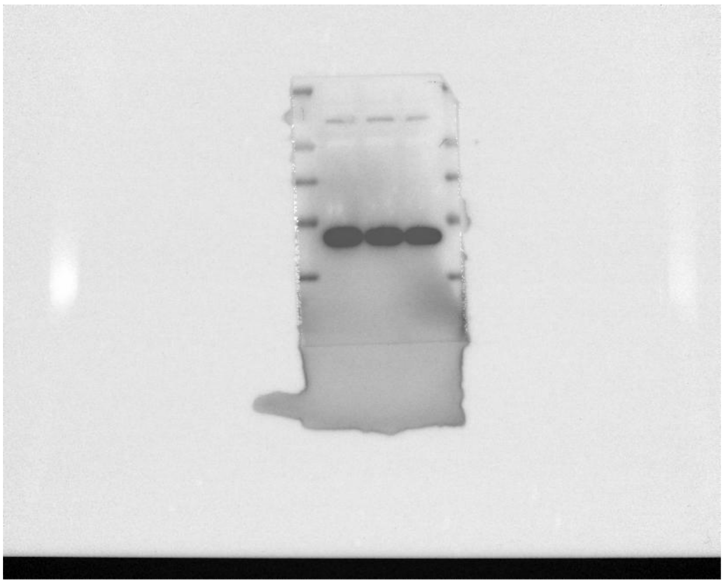

RAS

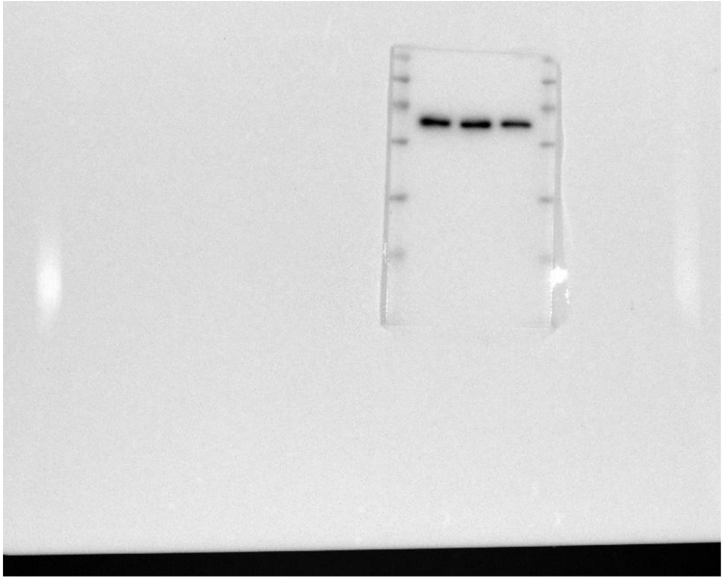

ERK

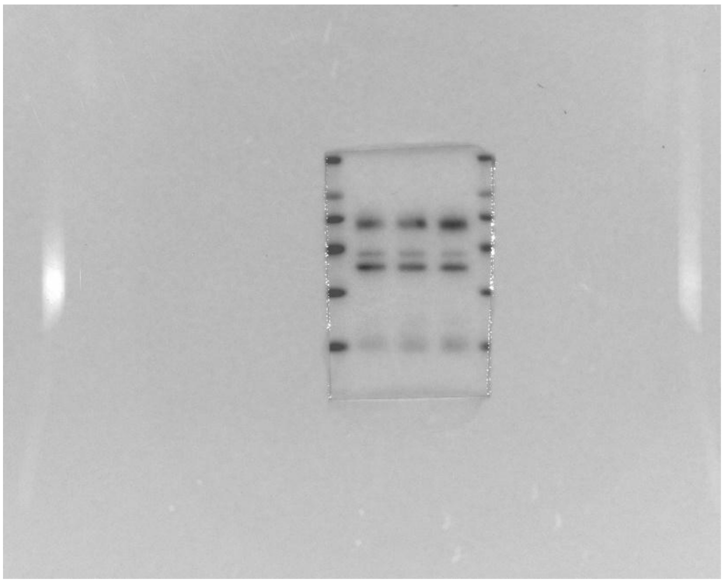

p-ERK

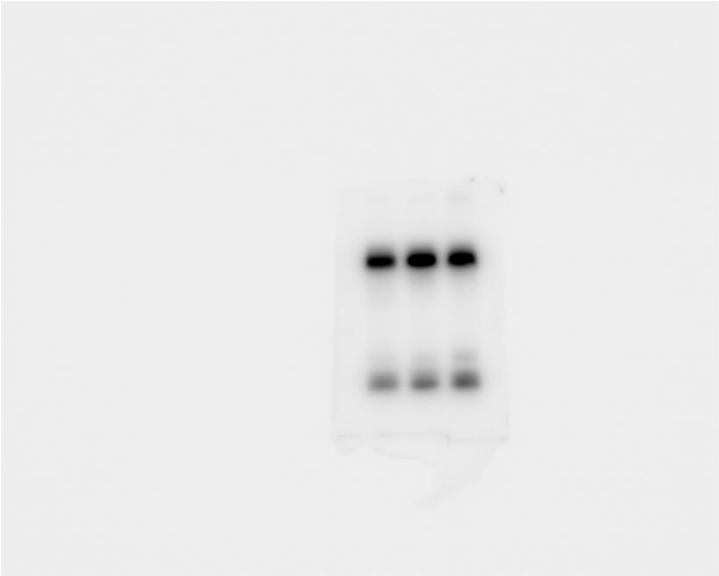

JNK

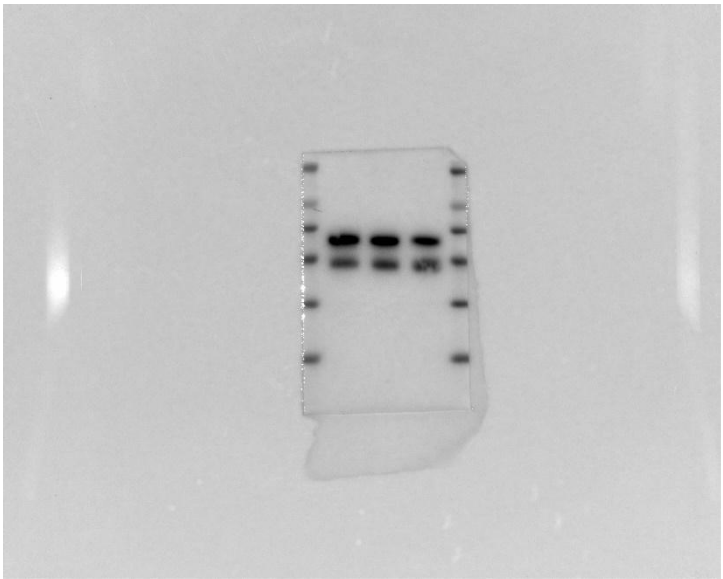

p-JNK

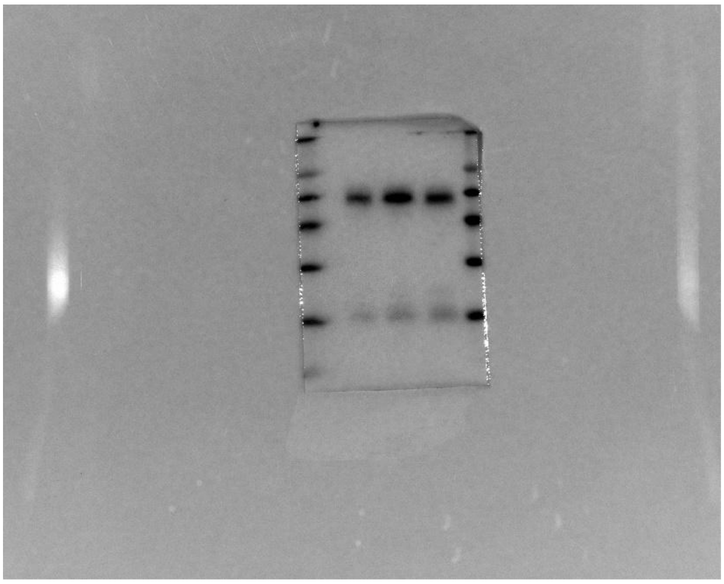

p38

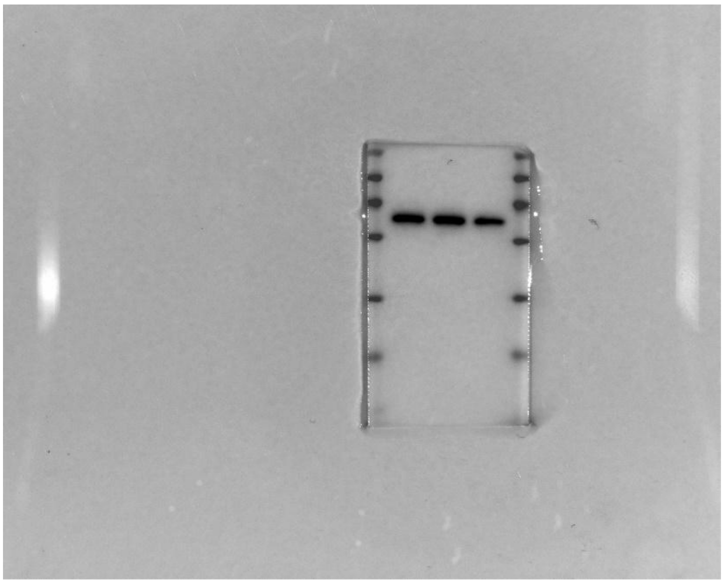

p-p38

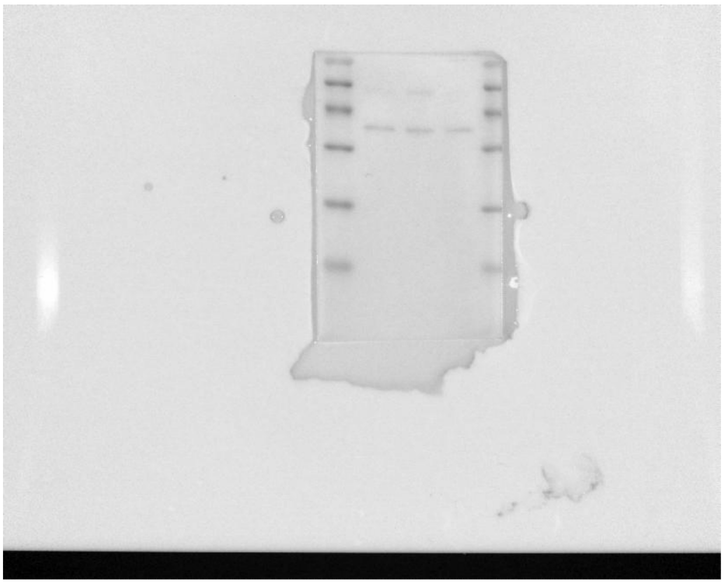

STAT3

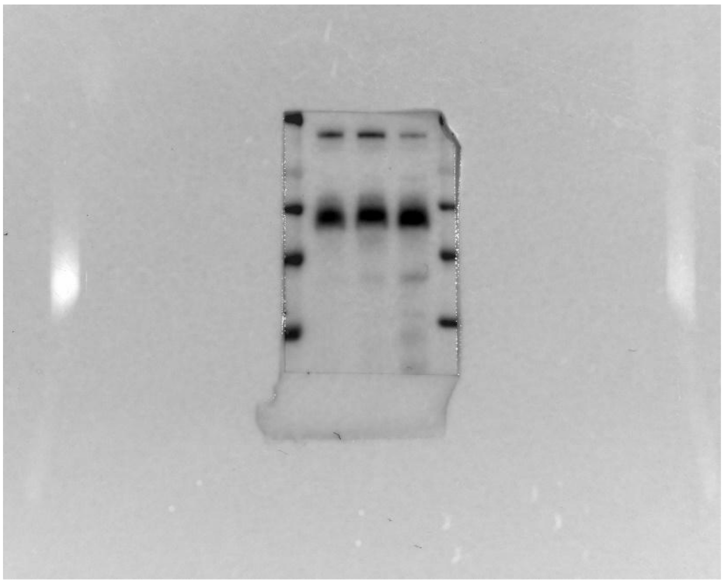

p-STAT3

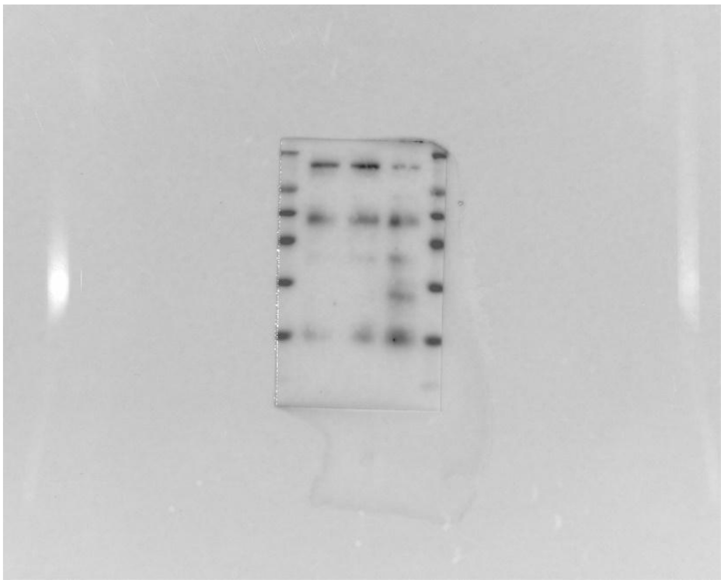

GAPDH

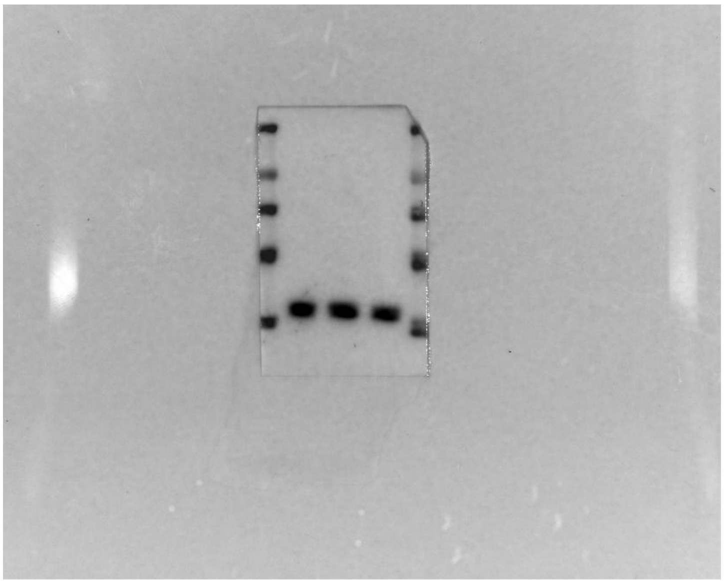

Figure6g

ERK

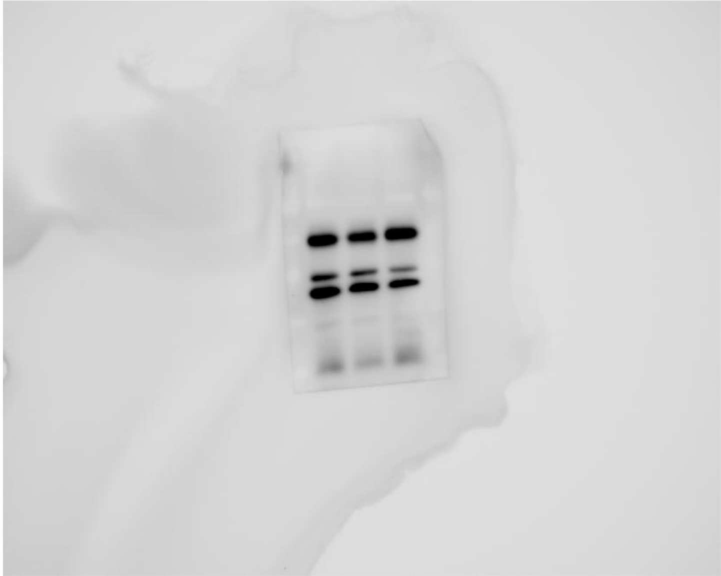

p-ERK

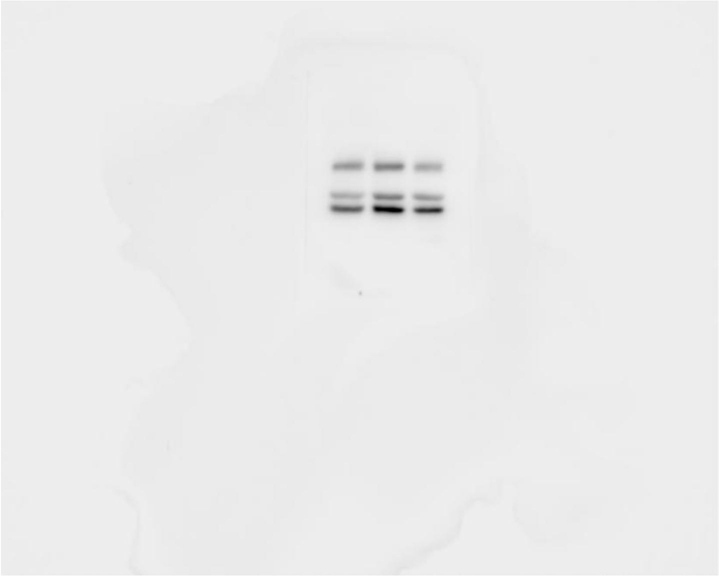

JNK

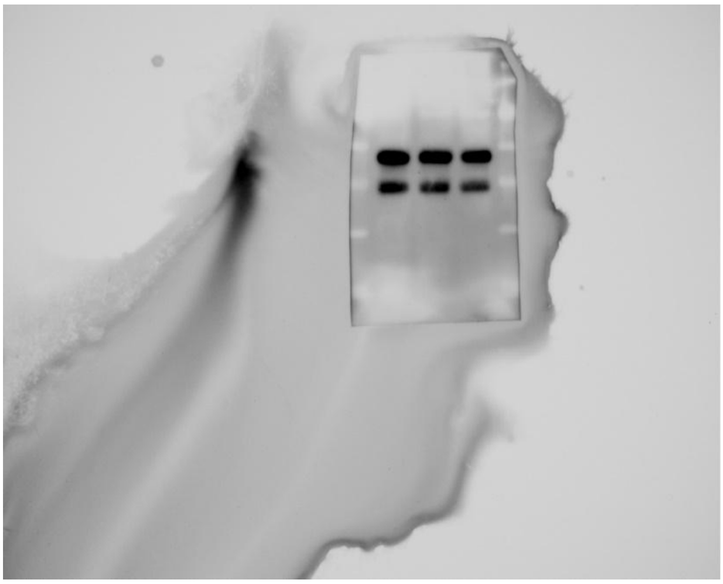

p-JNK

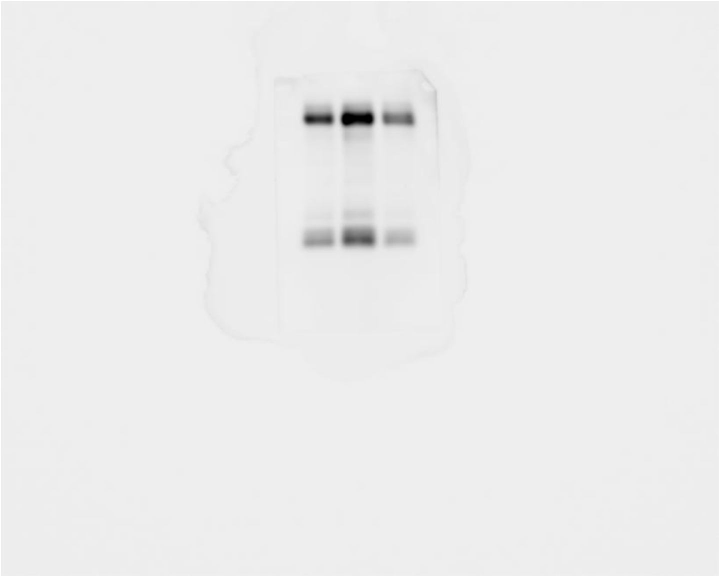

p38

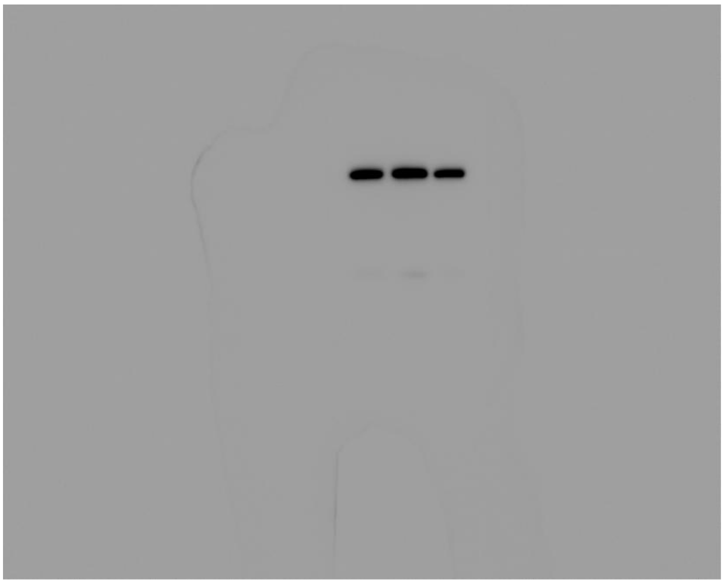

p-p38

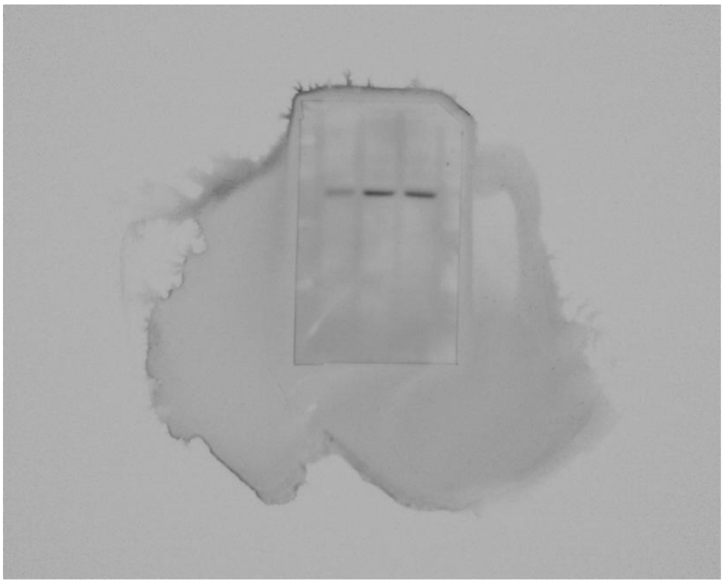

p-STAT3

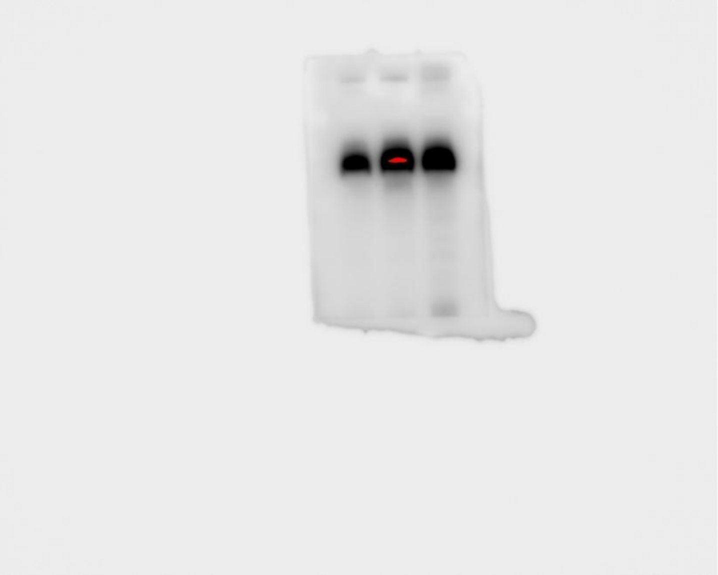

GAPDH

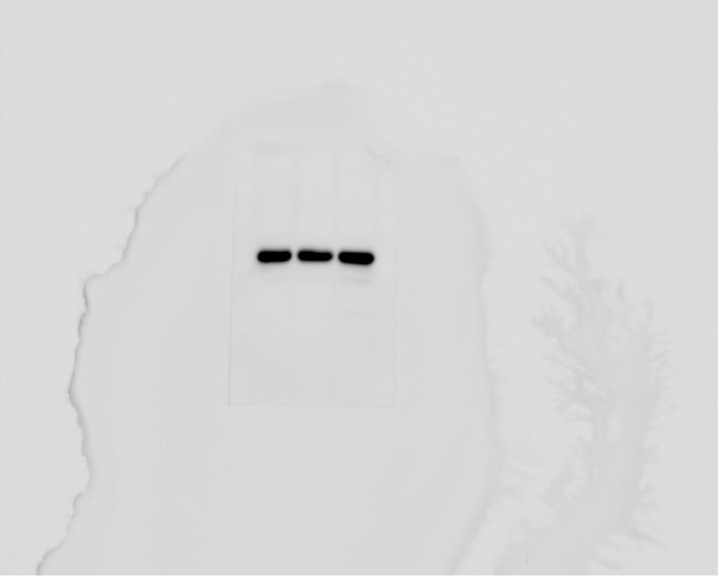

Figure6h

c-Fos

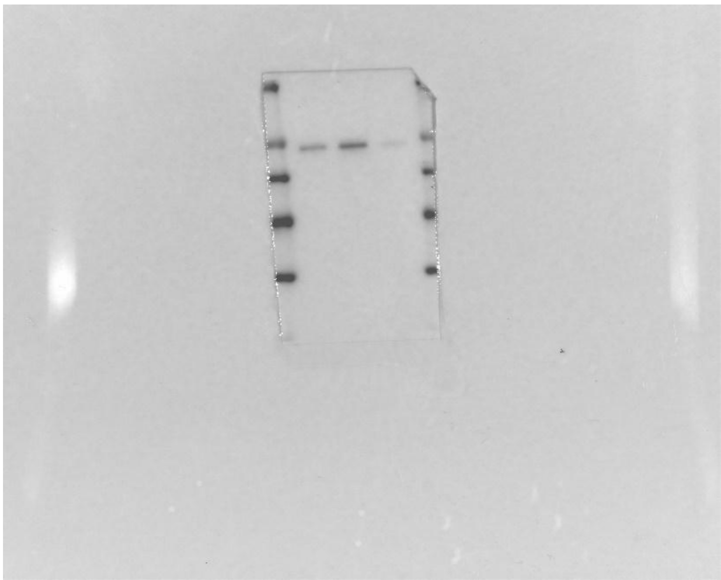

c-Jun

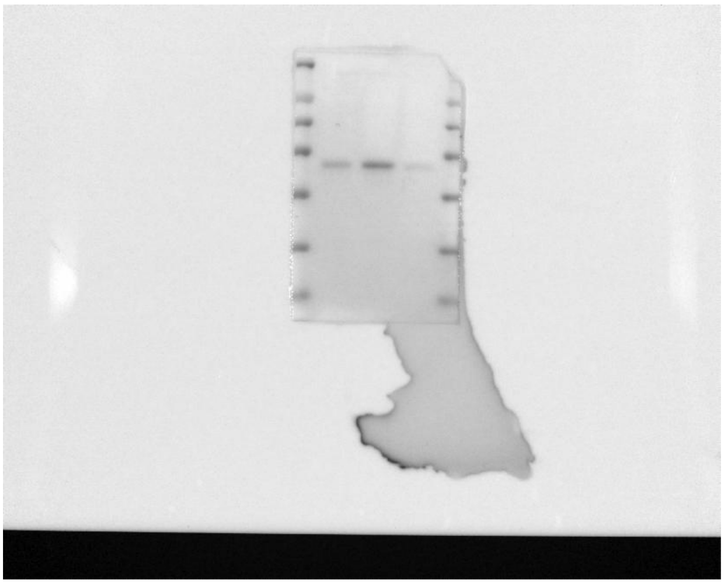

PCNA

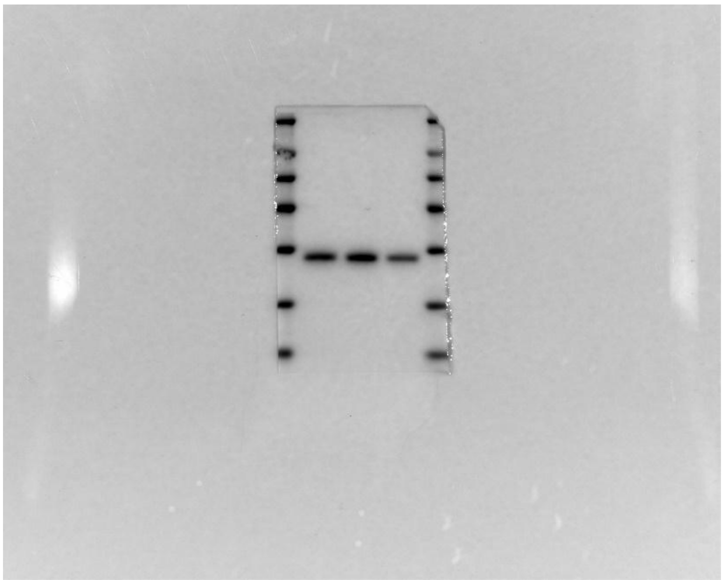

Supplement: Supplementary file 2 — Supplementary Information. [file 41598_2022_12695_MOESM2_ESM.pdf]
